# A randomized prospective study of neonatal hepatitis B vaccine immunogenicity in The Gambia and Papua New Guinea

**DOI:** 10.1016/j.jacig.2026.100653

**Published:** 2026-02-06

**Authors:** Joann Diray-Arce, Caitlin Syphurs, Annmarie Hoch, Kerry McEnaney, Kinga K. Smolen, Oghenebrume Wariri, Abdulazeez Imam, Tida Dibassey, Fatoumata Cole, Fatoumatta Ceesay, Alansana Darboe, William S. Pomat, Rebecca Ford, Geraldine Masiria, Dorcas Joseph, Joe Jude, Rym Ben-Othman, Casey P. Shannon, Sanya Thomas, Geert Leroux-Roels, Frédéric Clement, Gwenn Waerlop, Caitlyn McLoughlin, Sofia M. Vignolo, Shun Rao, Nelly Amenyogbe, The EPIC Consortium, Nelly Amenyogbe, Asimenia Angelidou, Winnie Bao, Rym Ben-Othman, Tue B. Bennike, Travis M. Blimke, Morten Bjerregaard-Andersen, Ryan R. Brinkman, Byron Brook, Kendyll Burnell, Bing Cai, Abhinav Checkervarty, Jing Chen, Virginia Chen, Mitchell Cooney, Momoudou Cox, Alansana Darboe, Bhavjinder K. Dhillon, Tida Dibassey, Joann Diray-Arce, Reza Falsafi, Benoit Fatou, Rebecca Ford, Freddy Francis, Christian N. Golding, Robert E.W. Hancock, Danny J. Harbeson, Daniel He, Samuel H. Hinshaw, Annmarie Hoch, Joe Huang, Olubukola T. Idoko, Abdulazeez Imam, Beate Kampmann, Meagan Karoly, Wendy Kirarock, Tobias R. Kollmann, Ken Kraft, Kristina Lindberg Larsen, Jessica Lasky-Su, Amy H. Lee, Ofer Levy, Aaron Liu, Mark Liu, Mehrnoush Malek, Arnaud Marchant, Geraldine Masiria, David Jim, John Paul Matlam, Kerry McEnaney, Caitlyn McLoughlin, Sebastiano Montante, Elena Morrocchi, Jorjoh Ndure, Athena Nguyen, Jainaba Njie-Jobe, Oludare A. Odumade, Al Ozonoff, Jensen Pak, Paolo Palma, Edward P.K. Parker, Matthew A. Pettengill, Alec Plotkin, William S. Pomat, Shun Rao, Peter C. Richmond, Elishia Roberts, Gerard Saleu, Lilica Sanca, Guzman Sanchez-Schmitz, Frederik Schaltz-Buchholzer, Casey P. Shannon, Amrit Singh, Maren Smith, Kinga K. Smolen, Hanno Steen, Julia Strandmark, Caitlin Syphurs, Scott J. Tebbutt, Anita H.J. van den Biggelaar, Simon D. van Haren, Natallia Varankovich, Sofia M. Vignolo, Diana Vo, Oghenebrume Wariri, Oludare Odumade, Asimenia Angelidou, Scott J. Tebbutt, Arnaud Marchant, Tobias R. Kollmann, Ofer Levy, Peter C. Richmond, Anita H.J. van den Biggelaar, Beate Kampmann, Olubukola T. Idoko, Al Ozonoff

**Affiliations:** a Precision Vaccines Program, Department of Pediatrics, Boston Children’s Hospital, Boston, Mass; b Department of Pediatrics, Harvard Medical School, Boston, Mass; c Broad Institute of MIT & Harvard, Cambridge, Mass; d Vaccines and Immunity Theme, Medical Research Council Unit, The Gambia at the London School of Hygiene and Tropical Medicine, Banjul, Gambia; e Papua New Guinea Institute of Medical Research, Goroka, Eastern Highlands Province, Papua New Guinea; f The Kids Research Institute Australia, Nedlands, Australia; g RAN BioLinks, Ltd, Toronto, Ontario, Canada; h PROOF Centre of Excellence, Providence Research, Vancouver, British Columbia, Canada; i BC Centre for Heart Lung Innovation, St. Paul’s Hospital, Vancouver, British Columbia, Canada; j Center for Vaccinology, Ghent University and Ghent University Hospital, Ghent, Belgium; k Department of Microbiology and Immunology, Division of Pediatrics, Dalhousie University, Halifax, Nova Scotia, Canada; l Department of Neonatology, Beth Israel Deaconess Medical Centre, Boston, Mass; m Department of Medicine, Division of Respiratory Medicine, University of British Columbia, Vancouver, British Columbia, Canada; n European Plotkin Institute for Vaccinology, Université libre de Bruxelles, Gosselies, Belgium; o Department of Microbiology and Immunology, University of British Columbia, Vancouver, British Columbia, Canada; p School of Medicine, The University of Western Australia, Crawley, Australia; q Centre for Child Health Research, The University of Western Australia, Crawley, Australia; r Vaccine Centre, Faculty of Infectious and Tropical Diseases, London School of Hygiene and Tropical Medicine, London, United Kingdom; s Centre for Global Health and Institute for International Health, Charité Universitätsmedizin, Berlin, Germany; t Department of Pathology, Dalhousie University, Halifax, Nova Scotia, Canada

**Keywords:** Vaccines, Expanded Program on Immunization, neonatal vaccination, hepatitis B vaccine, Bacille Calmette-Guérin, neonates, maternal antibodies, vaccine immunogenicity

## Abstract

**Background:**

Protection of newborns from infection can be achieved through maternal or vaccine-induced antibodies, but the factors influencing vaccine protection (correlate of protection) and subsequent infant immunity remain insufficiently understood. Further investigation is essential to optimize early-life vaccination strategies.

**Objective:**

We sought to evaluate the impact of timing and sequence of hepatitis B vaccine (HBV) and Bacille Calmette-Guérin (BCG) vaccine within the first week of life on infant HBV immunogenicity and its relationship with maternal antibodies.

**Methods:**

This was an exploratory analysis of a US National Institutes of Health–supported prospective, randomized study of systems biology signatures predictive of HBV immunogenicity in 2 geographically distinct cohorts in The Gambia (GAM; N = 720) and Papua New Guinea (PNG, N = 101) (clinicaltrials.gov NCT03246230). Healthy, hepatitis B surface antigen (anti-HBs)-negative mothers and their infants were enrolled and randomized into 4 vaccine groups: HBV alone, BCG alone, HBV + BCG, or no vaccination (delayed until no later than day of life [DOL]7). Blood samples were collected at birth (DOL0) and on a randomly assigned day (either DOL1, DOL3, or DOL7) with a maximum of 2 blood draws in the first week of life per ethics approval. All infants received catch-up vaccination within DOL7 and the recommended oral polio vaccine within 10 days of birth. Additional blood samples were collected at DOL30 and DOL128 to measure anti-HBs titers and assess immunogenicity.

**Results:**

Demographic and clinical characteristics were balanced across vaccine groups, suggesting successful randomization in both cohorts. Similar infant growth trajectories and longitudinal anti-HBs titer trends were observed across all vaccine groups in both cohorts over the first 128 days of life. Maternal and neonatal baseline anti-HBs titers were strongly correlated (GAM: *r* = 0.98; PNG: *r* = 0.99). DOL0 titers predicted DOL30 titers (GAM: *R*^2^ = 0.56; PNG: *R*^2^ = 0.945) but not DOL128 titers. Exploratory pairwise comparisons showed that Gambian infants receiving BCG at birth and HBV at DOL7 had higher DOL30 titers than those vaccinated with HBV at DOL1 (Wilcoxon *P* = .046), whereas in PNG, infants in the HBV + BCG group and the delayed group had higher DOL128 titers than those in the HBV group (Wilcoxon *P* = .046; Wilcoxon *P* = .019, respectively).

**Conclusions:**

This study demonstrates the feasibility of conducting large-scale neonatal immunogenicity studies in resource-constrained settings. Our observations underscore the importance of vaccine timing and maternal antibodies in shaping early-life vaccine-induced immunogenicity, offering valuable insights for neonatal vaccination schedules.

Infectious diseases are a leading cause of infant morbidity and mortality worldwide.^1^, ^2^, ^3^, ^4^ Vaccines significantly reduce this burden by protecting against life-threatening infections, with increased vaccination coverage leading to lower rates of vaccine-preventable diseases.^5^ The World Health Organization Expanded Program on Immunization (EPI) addresses this need by recommending early vaccination against key infections, including tuberculosis, hepatitis B, and poliomyelitis, during the highly vulnerable first month of life.

The human immune system undergoes age-dependent adaptations^6^ influencing vaccine efficacy. For example, the live-attenuated Bacille Calmette-Guérin (BCG) vaccine primes early-life immunity against unrelated pathogens, whereas subunit and polysaccharide vaccines achieve optimal immunogenicity later in life and following multiple doses.^7^ Another factor affecting neonatal vaccine response is the presence of transplacentally acquired maternal antibodies (MatAbs), providing passive protection during the infant’s first 6 months.^8^ However, MatAbs can interfere with the infant’s own humoral immune response to vaccination,^9^, ^10^, ^11^ reducing early antibody responses. To mitigate this interference, vaccination schedules are often delayed by several months after birth to allow the infant’s immune system to respond more effectively.^12^ The long-term effects of MatAbs on immunity are incompletely understood,^13^ and their influence on humoral vaccine responses necessitates careful consideration in immunization schedules.

The US National Institutes of Health/National Institutes of Allergy and Infectious Diseases–supported Expanded Program on Immunization Consortium (EPIC) primary study (EPIC-002) investigated early-life immunogenicity by analyzing peripheral blood samples from newborns before and after immunization with a licensed hepatitis B vaccine (HBV).^14^ The study aimed to define molecular signatures predictive of antibody titers against the hepatitis B surface antigen (anti-HBs). HBV was chosen for its well-established correlate of protection (CoP) against HBV infection, with anti-HBs levels greater than or equal to 10 mIU/mL recognized as a reliable measure of vaccine efficacy.^15^^,^^16^

Although anti-HBs titers greater than or equal to 10 mIU/mL are considered protective, the exact quantity of antibody titers correlates with both the level and duration of protection (ie, the higher the anti-HBs titer, the better and longer the protection).^16^ A robust antibody response after the first dose predicts sustained responses following the full vaccination series.^15^ Even when titers fall below 10 mIU/mL, most individuals who had initially achieved protective levels remain immune to HBV infection, suggesting antibody-independent mechanisms of protection such as immune memory mediated by antigen-specific B and T cells.^17^ Hepatitis B–specific T-cell immunity likely also contributes to long-term protection regardless of serum antibody levels.^18^^,^^19^

As part of EPI, the HBV was introduced into national immunization programs in The Gambia (GAM; 1990) and Papua New Guinea (PNG; 1989) to prevent perinatal transmission.^20^, ^21^, ^22^, ^23^ Despite these long-standing programs, timely birth-dose coverage remains variable in many settings due to limited access to facility-based deliveries and logistical challenges in vaccine delivery.^20^^,^^21^ Hepatitis B remains a public health concern in both regions, where perinatal and early childhood transmission still occur.^22^^,^^23^ These low-resource communities offer a unique opportunity to evaluate vaccine-induced immune responses under distinct health care contexts.

This report presents an exploratory analysis of clinical data from human newborn cohorts across 2 geographically distinct populations, in GAM and PNG, with the objective to evaluate the impact of timing and sequence of HBV and BCG vaccine within the first week of life on infant HBV immunogenicity and its relationship with MatAbs. These findings demonstrate the feasibility of this innovative study design and provide a foundation for systems biology analyses to identify biomarkers of neonatal vaccine immunogenicity, informing early-life vaccine development.

## Methods

### Study population

The US National Institutes of Health / National Institutes of Allergy and Infectious Diseases Human Immunology Project Consortium–supported EPIC-002 study consists of 2 prospective neonatal cohorts in GAM and PNG. The protocols were harmonized and previously described.^14^ The GAM cohort enrolled mother-infant pairs at Banjulinding Health Centre and Kanifing General Hospital (September 2017-June 2019), whereas the validation cohort from PNG was enrolled at Goroka Hospital (September 2020-April 2022; see Fig E1 in this article’s Online Repository at www.jaci-global.org). Study details were presented during community meetings, and individual sensitization occurred in antenatal clinics.^24^ Written informed consent was obtained from parents before enrollment, either shortly before or after delivery. Ethical approval for the Gambian cohort was granted by the Gambian Government / Medical Research Council Gambia Joint Ethics Committee (Scientific Coordinating Committee no. 1513) and Boston Children’s Hospital Institutional Review Board (IRB-P00024239), and for PNG, PNG Institute of Medical Research IRB (IRB#1812) and the PNG Medical Research Advisory Committee (MRAC#18.24). The protocol is registered at clinicaltrials.gov (NCT03246230; https://clinicaltrials.gov/study/NCT03246230).

### Sample collection and study design

Peripheral blood samples were collected from anti-HBs–negative mothers with no history of tuberculosis diagnosis at the time of delivery. Baseline samples from newborns were collected on day of life (DOL)0, immediately after birth and before vaccination, during visit 1 (V1). Infants were randomized into 4 vaccine groups to receive at birth either (1) HBV alone, (2) BCG alone, (3) HBV + BCG, or (4) no vaccination (delayed until after visit 2 [V2] but no later than DOL7). For V2, peripheral blood samples were collected on either DOL1, DOL3, or DOL7 based on subgroup randomization, limiting sampling to 2 blood draws in the first week of life per ethics approval.^14^ Regardless of group assignment, participants received catch-up vaccination after V2, no later than DOL7. All participants received the recommended birth dose of the oral polio vaccine within 10 days postbirth.

Visit 3 (V3) occurred on DOL30 and visit 4 (V4) on DOL128, 30 days after completing the primary HBV immunization series (3 doses). Infants in the delayed group did not contribute to V3 or V4 data because they received their catch-up vaccinations during their first week of life and no longer served as non–vaccine-induced controls at these time points. The PNG validation cohort followed the same study design, except that V2 samples were taken on DOL7 only, and the delayed group contributed to V3 and V4 to assess the effect of delayed vaccination on immunogenicity in this cohort. All participants received the World Health Organization–recommended EPI schedule vaccines according to protocol.^14^ Of note, the routine schedule in GAM entails vaccinations at age 2, 3, and 4 months beyond the birth doses whereas that of PNG is 1, 2, and 3 months.^14^

### Anti-HBs antibody titers

Anti-HBs titers were measured at the Centre for Vaccinology, Ghent University, Belgium, using the Architect (or equivalent Alinity) analyzer (Abbott Laboratories; Chicago, Ill) following manufacturer’s instructions (kit ref: 7C18). The assay had an analytical range of 2.50 to 1000.00 mIU/mL, with Abbott-provided negative and positive controls included in each run. For samples with insufficient volume, a dilution was performed using FCS, and an additional FCS-based negative control was included in the run. Universal Reference Standards (ie, pooled plasma from newborn cord blood) were also included in each run to minimize run-to-run variation. Samples with volumes less than 10 μL were flagged as Quantity Not Sufficient and excluded. Titers higher than 1000.00 mIU/mL were diluted 1:100 with FCS and retested, whereas titers below 2.50 mIU/mL were reported as less than 2.50 mIU/mL. Anti-HBs concentrations were imputed on the basis of lower limit of quantification and log-transformed for analysis.

### Data management

Data collected included participant identification, randomization details, eligibility criteria, confirmation of informed consent, demographic information, vital signs, growth measurements, physical examinations, adverse events, vaccination history, medications, birth delivery details, sample collection, and laboratory investigations.^14^ Clinical laboratory and diagnostic data were added as available on the basis of infant’s clinical status. The Data Management Core at Boston Children’s Hospital used a secure, cloud-based bioinformatic architecture to securely curate, store, and analyze multicenter data sets in real time.^25^ Biological samples, sample metadata, and clinical data were tracked via *ItemTracker* and REDCap, with rigorous quality control and quality assurance processes ensuring data integrity. Importantly, this occurred during the active participant enrollment period to ensure rapid responsiveness from the clinical staff on site, given the quick turnover of personnel after enrollment. Finalized, deidentified, quality-assessed data sets were deposited into public repositories such as *ImmPort* (SDY1538; SDY2584), facilitating timely data sharing with the broader research community.

### Statistical analysis

Statistical analyses were conducted on the GAM cohort and validated with the PNG cohort (R version 4.1.2; *α* < 0.05). Demographic differences across vaccine groups were assessed using chi-square tests, whereas infant growth measures and anti-HBs titers were compared using the Kruskal-Wallis rank-sum test and Wilcoxon pairwise comparisons. Longitudinal trajectories in mean growth measures were analyzed (DOL30/DOL0; DOL128/DOL0). Paired maternal-infant anti-HBs titers at DOL0 were assessed using Pearson correlation, and univariate linear regression models estimated relationships between DOL0 anti-HBs titers and infant anti-HBs titers at DOL30 and DOL128. To address variability in anti-HBs assays below the CoP,^26^ we subset paired maternal-infant anti-HBs titer samples for concordant measures of detectable (≥2.5 mIU/mL) DOL0 anti-HBs titers. This excluded 83 (12.4%) paired GAM maternal-infant samples and 1 (1.9%) paired PNG maternal-infant sample, leaving 589 (87.6%) paired GAM maternal-infant samples and 52 (98.1%) paired PNG maternal-infant samples for analysis. Detailed Pearson correlation results are provided (see Fig E2 in this article’s Online Repository at www.jaci-global.org). The cohort design included careful consideration of statistical power and sample size, and a summary of power calculations performed has been previously published.^14^

## Results

### Study cohort

**Fig 1:**
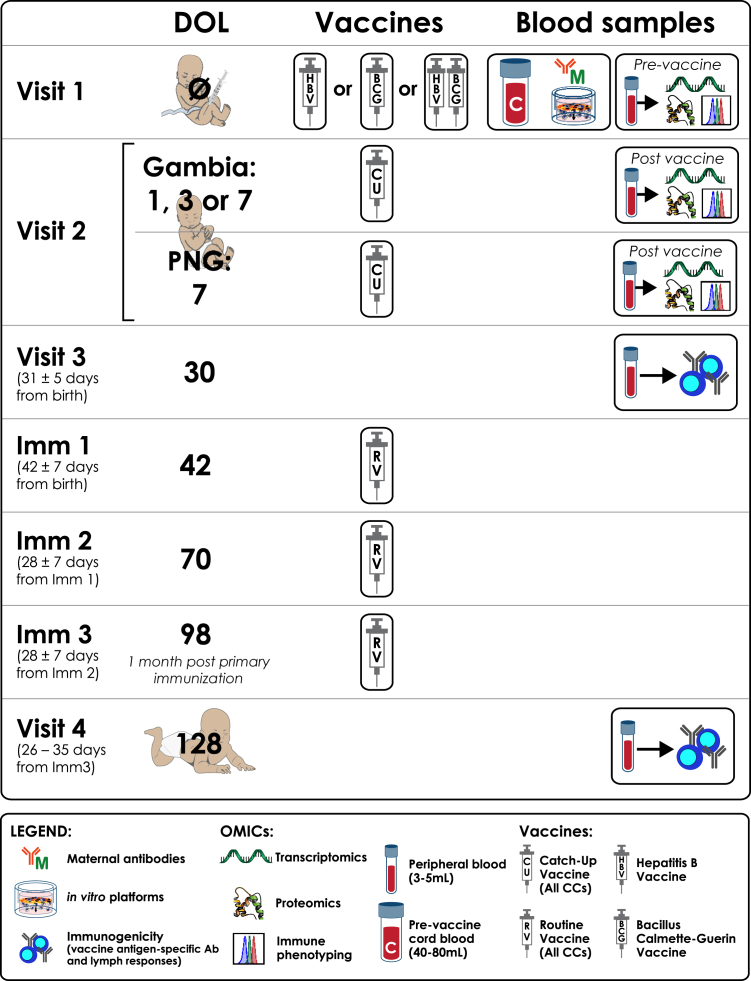
EPIC GAM and PNG validation cohort design. Study design graphic depicting the EPIC-002 study protocol, including randomization groups and blood sample collection time points. *Ab*, Antibdoy; *Imm*, immunization.

**Fig 2:**
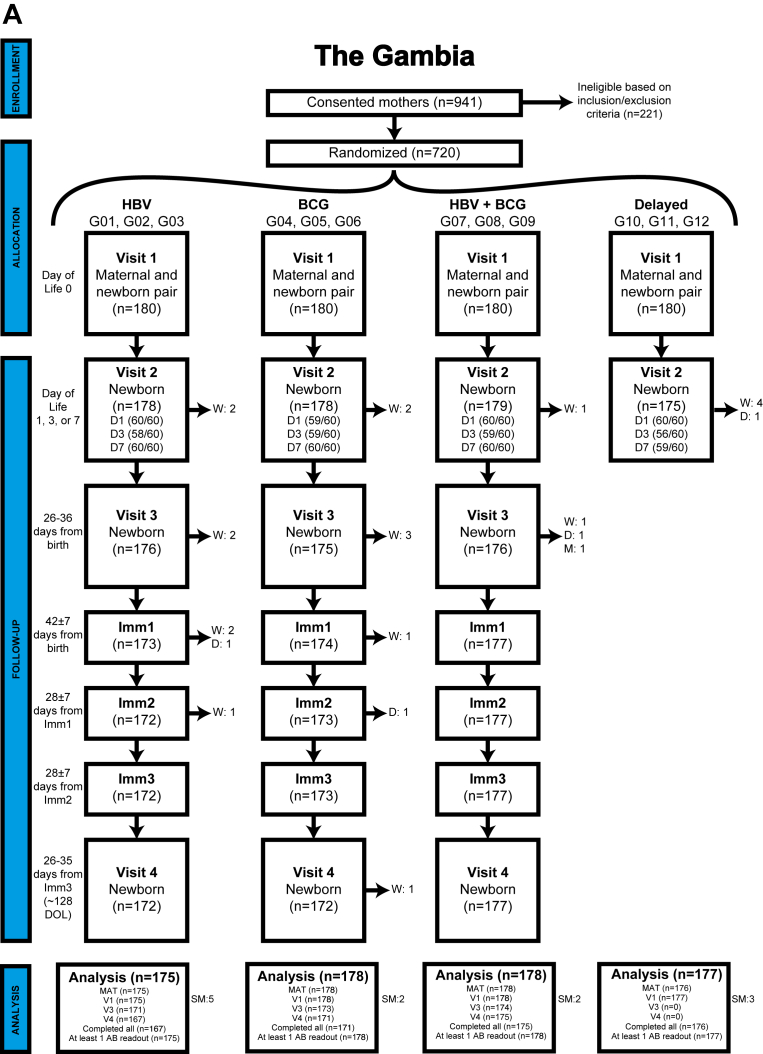

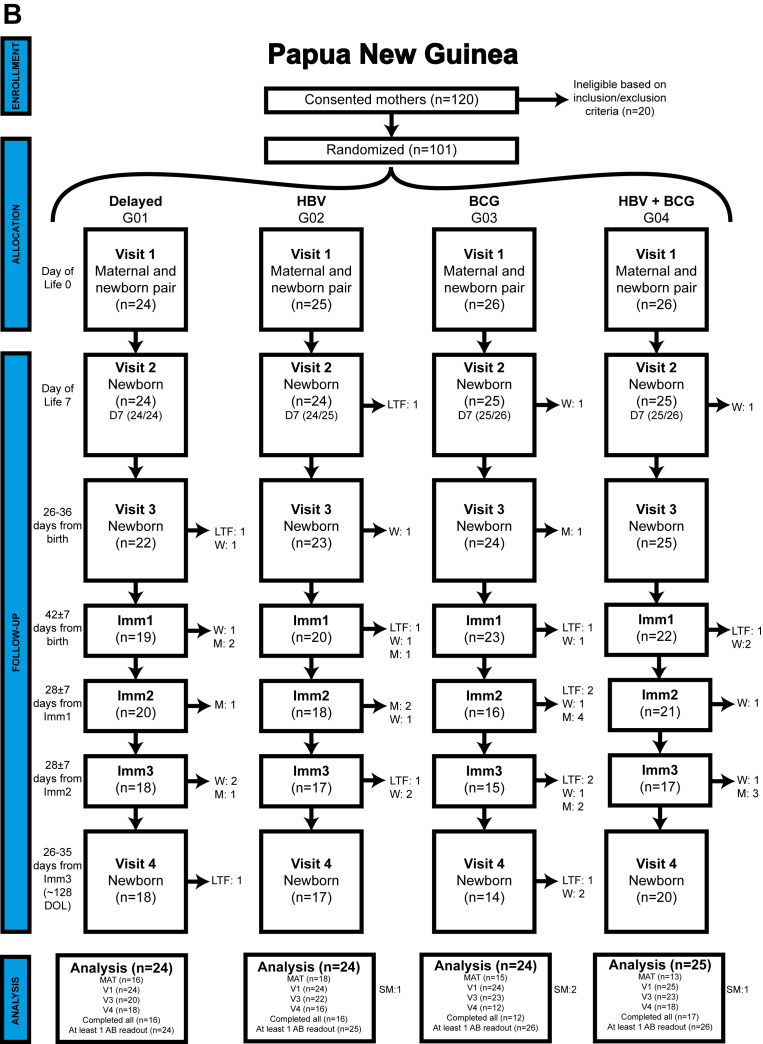
CONSORT diagrams. (**A**) GAM and (**B**) PNG validation cohorts (AB = antibody; LTF = lost to follow-up, W = withdrawn, D = died, M = missing, SM = sex-mismatch). The PNG validation cohort included a maternal-infant pair of a set of twins. Therefore 100 mothers were eligible to participate, with 101 infants randomized to the vaccine groups.

The EPIC-002 study characterizes vaccine-induced systems biology signatures and their relationship to HBV CoP, as well as the impact of BCG and MatAbs, to define biomarkers predictive of neonatal vaccine immunogenicity and inform early-life vaccine development. The overall EPIC-002 study design, including visit schedules, vaccinations, and blood sample collections for GAM and PNG cohorts, is detailed (Fig 1) with enrollment, allocation, follow-up, and analysis flow depicted (Fig 2). For this exploratory report, only clinical and vaccine antigen-specific antibody data in neonates were assessed.

In the Gambian cohort, 941 mothers consented to participate, with 221 found ineligible based on exclusion criteria,^14^ resulting in enrollment and randomization of 720 (77%) mother-infant pairs into immunization groups (Fig 2, *A*). Of the 720 randomized mother-infant pairs, 180 (25.0%) infants in the delayed group were not followed to V3 and V4. Among the remaining 540 (75.0%) participants in the HBV, BCG, or HBV + BCG group, 521 (96.5%) had samples available at V4. Only 19 (3.5%) GAM infants lacked V4 samples due to consent withdrawal (N = 16; 84.2%), or death before V4 (N = 3; 15.8%) (Fig 2, *A*).

In the PNG cohort, 120 mothers consented, with 20 excluded on the basis of eligibility criteria. The final PNG cohort included 101 eligible infant participants (from 100 mothers comprising 99 single births and 1 set of twins), with 69 (68.3%) having V4 samples available (Fig 2, *B*). Unlike the GAM cohort, PNG infants in the delayed group were followed to V4; however, 32 (31.7%) infants were missing V4 samples due to consent withdrawal (20 [62.5%]) and loss to follow-up (12 [37.5%]) (Fig 2, *B*). Rigorous quality control / quality assurance protocols^25^ confirmed clinical variables, including biological sex consistency with transcriptomics and epigenetic sex determination analyses. After screening, 708 (98.3%) Gambian participants and 97 (96.0%) PNG participants were deemed eligible for analysis.

### Clinical and demographic cohort characteristics

Baseline characteristics of the GAM and PNG maternal-infant cohorts across vaccine groups are presented (Tables I and II). Among GAM mothers, 47.0% were aged between 26 and 35 years, and 47.6% belonged to the Mandinka ethnicity (Table I). The PNG cohort had a larger proportion of mothers (52.6%) aged between 17 and 25 years, with 86.6% belonging to an Eastern Highland ethnicity (Table II).

**Table I:** Baseline characteristics of Gambian infant cohort

Characteristic	Total (N = 708)	HBV (N = 175)	BCG (N = 178)	HBV + BCG (N = 178)	Delayed (N = 177)
Sex, n (%)					
Male	362 (51.1)	90 (51.4)	88 (49.4)	91 (51.1)	93 (52.5)
Female	346 (48.9)	85 (48.6)	90 (50.6)	87 (48.9)	84 (47.5)
Ethnicity, n (%)					
Fula	86 (12.1)	28 (16.0)	22 (12.4)	18 (10.1)	18 (10.2)
Jola	110 (15.5)	27 (15.4)	25 (14.0)	30 (16.9)	28 (15.8)
Mandinka	337 (47.6)	85 (48.6)	88 (49.4)	90 (50.6)	74 (41.8)
Serahule	28 (4.0)	8 (4.6)	5 (2.8)	9 (5.1)	6 (3.4)
Wollof	80 (11.3)	16 (9.1)	21 (11.8)	13 (7.3)	30 (16.9)
Others	67 (9.5)	11 (6.3)	17 (9.6)	18 (10.1)	21 (11.9)
Birth season, n (%)					
Dry season (November-May)	468 (66.1)	118 (67.4)	115 (64.6)	119 (66.9)	116 (65.5)
Rainy season (June-October)	240 (33.9)	57 (32.6)	63 (35.4)	59 (33.1)	61 (34.5)
Estimated gestational age (wk), n (%)					
Early-term (36-38)	181 (25.6)	41 (23.4)	46 (25.8)	48 (27.0)	46 (26.0)
Full-term (39-40)	437 (61.7)	114 (65.1)	108 (60.7)	109 (61.2)	106 (59.9)
Late-term (>41)	90 (12.7)	20 (11.4)	24 (13.5)	21 (11.8)	25 (14.1)
Maternal hepatitis B antibody status, n (%)					
Nondetectable	383 (54.1)	100 (57.1)	100 (56.2)	100 (56.2)	83 (46.9)
Detectable	294 (41.5)	65 (37.1)	71 (39.9)	71 (39.9)	87 (49.2)
Missing	31 (4.4)	10 (5.7)	7 (3.9)	7 (3.9)	7 (4.0)
Breast-feeding (V1), n (%)					
Yes	627 (88.6)	143 (81.7)	165 (92.7)	158 (88.8)	161 (91.0)
No	81 (11.4)	32 (18.3)	13 (7.3)	20 (11.2)	16 (9.0)
Maternal age (y), n (%)					
17-25	234 (33.1)	60 (34.3)	61 (34.3)	53 (29.8)	60 (33.9)
26-35	333 (47.0)	83 (47.4)	79 (44.4)	88 (49.4)	83 (46.9)
>35	141 (19.9)	32 (18.3)	38 (21.3)	37 (20.8)	34 (19.2)
Infant hospitalized, n (%)					
Yes	59 (8.3)	20 (11.4)	12 (6.7)	16 (9.0)	11 (6.2)
No	649 (91.7)	155 (88.6)	166 (93.3)	162 (91.0)	166 (93.8)
5-minute APGAR score, median [min-max]	10.0 [8.0-10.0]	10.0 [8.0-10.0]	10.0 [8.0-10.0]	10.0 [8.0-10.0]	10.0 [8.00-10.0]
Birth weight (g), mean ± SD	3160 ± 384	3150 ± 382	3150 ± 373	3200 ± 407	3140 ± 375
Length (cm), mean ± SD	48.7 ± 2.12	48.7 ± 2.02	48.8 ± 2.16	49.0 ± 2.06	48.4 ± 2.22
Head circumference (cm), mean ± SD	34.1 ± 1.29	34.1 ± 1.30	34.1 ± 1.24	34.2 ± 1.35	34.1 ± 1.27
Temperature (Celsius), median [min-max]	36.0 [34.0-37.0]	36.0 [34.0-37.0]	36.0 [35.0-37.0]	36.0 [35.0-37.0]	36.0 [34.0-37.0]
Missing, n (%)	2 (0.3)	0 (0)	0 (0)	1 (0.6)	1 (0.6)
Respiratory rate (cycles/min), median [min-max]	48.0 [24.0-89.0]	48.0 [34.0-81.0]	48.0 [29.0-89.0]	48.0 [24.0-77.0]	48.0 [38.0-68.0]
Heart rate (beats/min), median [min-max]	134 [92.0-179]	133 [95.0-172]	133 [102-161]	134 [95.0-176]	134 [92.0-179]

**Table II:** Baseline characteristics of PNG infant cohort

Characteristics	Total (N = 97)	HBV (N = 24)	BCG (N = 24)	HBV + BCG (N = 25)	Delayed (N = 24)
Sex, n (%)					
Male	47 (48.5)	14 (58.3)	11 (45.8)	12 (48.0)	10 (41.7)
Female	50 (51.5)	10 (41.7)	13 (54.2)	13 (52.0)	14 (58.3)
Ethnicity, n (%)					
Eastern Highland	84 (86.6)	23 (95.8)	22 (91.7)	19 (76.0)	20 (83.3)
Other	13 (13.4)	1 (4.2)	2 (8.3)	6 (24.0)	4 (16.7)
Birth season, n (%)					
Dry season (May-October)	39 (40.2)	8 (33.3)	9 (37.5)	12 (48.0)	10 (41.7)
Wet season (November-April)	58 (59.8)	16 (66.7)	15 (62.5)	13 (52.0)	14 (58.3)
Estimated gestational age (wk), n (%)					
Early-term (36-38)	65 (67.0)	14 (58.3)	16 (66.7)	17 (68.0)	18 (75.0)
Full-term (39-40)	31 (32.0)	9 (37.5)	8 (33.3)	8 (32.0)	6 (25.0)
Late-term (>41)	1 (1.0)	1 (4.2)	0 (0)	0 (0)	0 (0)
Maternal hepatitis B antibody status, n (%)					
Detectable	39 (40.2)	12 (50.0)	6 (25.0)	8 (32.0)	13 (54.2)
Nondetectable	53 (54.6)	12 (50.0)	17 (70.8)	15 (60.0)	9 (37.5)
Missing	5 (5.2)	0 (0)	1 (4.2)	2 (8.0)	2 (8.3)
Breast-feeding (V1), n (%)					
Yes	96 (99.0)	24 (100)	24 (100)	24 (96.0)	24 (100)
No	1 (1.0)	0 (0)	0 (0)	1 (4.0)	0 (0)
Maternal age (y), n (%)					
17-25	51 (52.6)	11 (45.8)	12 (50.0)	14 (56.0)	14 (58.3)
26-35	36 (37.1)	10 (41.7)	11 (45.8)	8 (32.0)	7 (29.2)
>35	10 (10.3)	3 (12.5)	1 (4.2)	3 (12.0)	3 (12.5)
5-min APGAR score, median [min-max]	10.0 [8.00-10.0]	10.0 [8.00-10.0]	10.0 [8.00-10.0]	10.0 [8.00-10.0]	10.0 [8.00-10.0]
Birth weight (g), mean ± SD	3330 ± 447	3340 ± 397	3490 ± 496	3230 ± 460	3250 ± 402
Length (cm), mean ± SD	46.4 ± 4.25	45.6 ± 5.77	47.1 ± 3.28	46.1 ± 3.44	47.0 ± 4.13
Head circumference (cm), mean ± SD	34.9 ± 2.36	34.7 ± 1.85	35.7 ± 2.66	35.0 ± 2.84	34.1 ± 1.75
Temperature (Celsius), median [min-max]	36.0 [35.8-36.7]	36.0 [35.9-36.5]	36.1 [35.8-36.7]	36.0 [35.8-36.6]	36.0 [35.8-36.7]
Respiratory rate (cycles/min), median [min-max]	50.0 [30.0-58.0]	50.0 [34.0-56.0]	48.0 [30.0-58.0]	49.0 [38.0-56.0]	50.0 [38.0-54.0]
Heart rate (beats/min), median [min-max]	146 [128-156]	146 [134-156]	142 [128-152]	146 [128-154]	148 [128-153]

In the GAM cohort, 51.1% of the infants were male, with 66.1% born during the Gambian dry season (November-May). Based on estimated gestational age, assessed using the modified Ballard score, 181 (25.6%) newborns were early-term (36-38 weeks), 437 (61.7%) full-term (39-40 weeks), and 90 (12.7%) late-term (≥41 weeks). APGAR scores at 5 minutes ranged between 8 and 10, and birth anthropometrics included a mean birth weight of 3160 g, mean birth length of 48.7 cm, and mean birth head circumference of 34.1 cm (Table I). There were 59 (8.3%) Gambian infants hospitalized during follow-up, of which 20 (33.9%) were randomized to the HBV group, 12 (20.3%) to the BCG group, 16 (27.1%) to the HBV + BCG group, and 11 (18.6%) to the delayed group (Table I; see Table E1 in this article’s Online Repository at www.jaci-global.org).

In the PNG cohort, female newborns comprised 51.5% of the cohort, with 58 (59.8%) infants born during the PNG wet season (November-April). According to estimated gestational age, 65 (67.0%) newborns were early-term (36-38 weeks), 31 (32.0%) full-term (39-40 weeks), and 1 (1.0%) late-term (≥41 weeks). APGAR scores at 5 minutes ranged between 8 and 10, mean birth weight was 3330 g, mean birth length was 46.4 cm, and mean birth head circumference was 34.9 cm (Table II). No hospitalization records were available for the PNG cohort due to restrictions during the 2020 COVID-19 pandemic.

Most GAM newborns (88.6%) were breast-fed soon after delivery and by V2, only 2 infants were not breast-fed (Table I). In the PNG cohort, only 1 infant (1.0%) was not breast-feeding at the conclusion of V1 (Table II). All other demographic and clinical characteristics were balanced across vaccine groups, suggesting successful randomization in both cohorts.

No significant differences across vaccine groups were observed in mean infant growth measures for head circumference, length, and weight at DOL30 and DOL128 in the GAM cohort (see Fig E3 in this article’s Online Repository at www.jaci-global.org). PNG infants had similar longitudinal growth measures; however, a significant difference in mean weight was observed at DOL128 (Kruskal-Wallis *P* = .02), in which infants in the BCG vaccine group had a higher mean weight than infants in the delayed group (Wilcoxon *P* = .001, *r* = 0.56) (see Fig E3 and Fig E4, *B*, in this article’s Online Repository at www.jaci-global.org). No significant differences were observed across vaccine groups when comparing the change in infant growth measures at follow-up visits compared with baseline (DOL30/DOL0; DOL128/DOL0) (Fig E4, *D*). Overall, infants in both cohorts demonstrated healthy growth trajectories over the first 128 days of life.

### Differences in infant anti-HBs titers across vaccine groups

**Fig 3:**
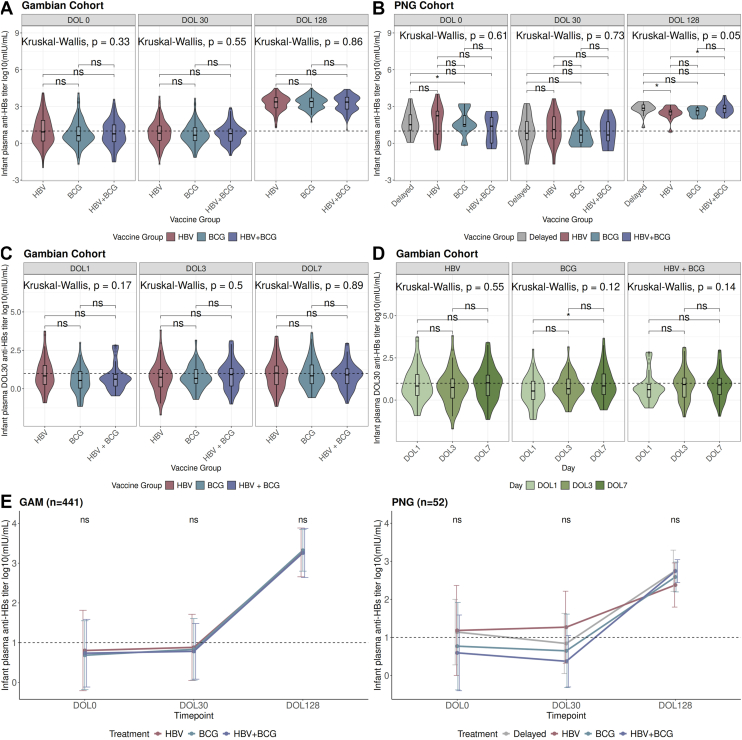
Infant anti-HBs titer response across vaccine and day randomization groups. Kruskal-Wallis rank-sum and Wilcoxon pairwise comparison significance markers indicating differences in anti-HBs titers assessed in (**A**) Gambian and (**B**) PNG infants across vaccine groups (HBV, BCG, HBV + BCG, delayed) at each time point (DOL0, DOL30, DOL128) (ns = not significant, ∗<.05). The delayed vaccine group was only followed to DOL30 and DOL128 time points in PNG infants. Kruskal-Wallis and Wilcoxon pairwise comparison *P* values indicating (**C**) differences in DOL30 anti-HBs titer response based on timing of catch-up vaccination at V2 in Gambian infants stratified by vaccine groups (HBV, BCG, HBV + BCG) and (**D**) differences in DOL30 anti-HBs titer response based on vaccine groups (HBV, BCG, HBV + BCG) in Gambian infants stratified by V2 catch-up vaccination time points. These analyses were performed on maternal-infant pairs with concordant detectable (≥2.5 mIU/mL) and nondetectable (<2.5 mIU/mL) anti-HBs titers at DOL0. The dotted horizontal line represents the CoP (10 mIU/mL) for anti-HBs titers. **E,** Kruskal-Wallis rank-sum significance markers comparing mean anti-HBs titers assessed in infants across vaccine groups (HBV, BCG, HBV + BCG, delayed) at each time point (DOL0, DOL30, DOL128) in Gambian and PNG infants with concordant detectable (≥2.5 mIU/mL) and nondetectable (<2.5 mIU/mL) anti-HBs titers at DOL0 who had paired samples across all time points (GAM n = 441; PNG n = 52). Mean log_10_ anti-HBs titers and SE bars are shown for each vaccine group at each time point. The delayed vaccine group was only followed to DOL30 and DOL128 time points in PNG infants. This analysis was performed on maternal-infant pairs. The dotted horizontal line represents the CoP (10 mIU/mL) for anti-HBs titers (ns = not significant, ∗<.05).

No significant differences in GAM anti-HBs titers were observed across vaccine groups at each visit in both cohorts (Fig 3, *A* and *B*). However, exploratory pairwise comparisons suggested that PNG infants in the HBV vaccine group had lower DOL128 anti-HBs titers compared with infants in the delayed (Wilcoxon *P* = .019, *r* = 0.41) and HBV + BCG (Wilcoxon *P* = .046, r = 0.34) vaccine groups (Fig 3, *B*). Furthermore, PNG infants in the BCG vaccine group had higher DOL0 anti-HBs titers than infants in the delayed group (Fig 3, *B*; Wilcoxon *P* = .037, *r* = 0.081); however, the overall Kruskal-Wallis test did not reach significance (*P* = .061). Overall, we observed similar patterns among longitudinal infant anti-HBs titers from DOL0 to DOL128 across all vaccine groups in both cohorts (Fig 3, *E*).

When looking at timing of vaccination on DOL30 GAM infant anti-HBs titers, we found no significant differences across vaccine groups (Fig 3, *C*). However, although the global test did not reach significance (Kruskal-Wallis *P* = .12), exploratory pairwise comparisons suggested that GAM infants who received BCG vaccination at birth and HBV at DOL7 had higher DOL30 titers, compared with infants who received BCG at birth and HBV at DOL1 (Wilcoxon *P* = .046) (Fig 3, *D*). When comparing infants with BCG versus HBV + BCG vaccination at birth, there were no significant differences observed in DOL30 anti-HBs titers (Wilcoxon *P* = .932) (Fig 3, *C*). By DOL128, all infants in both cohorts reach the CoP, irrespective of vaccine treatment (Fig 3, *E*). All delayed vaccines were given at DOL7 per PNG protocol, precluding validation of these findings in the PNG cohort.

### Impact of maternal anti-HBs titers on infant anti-HBs titer responses

**Fig 4:**
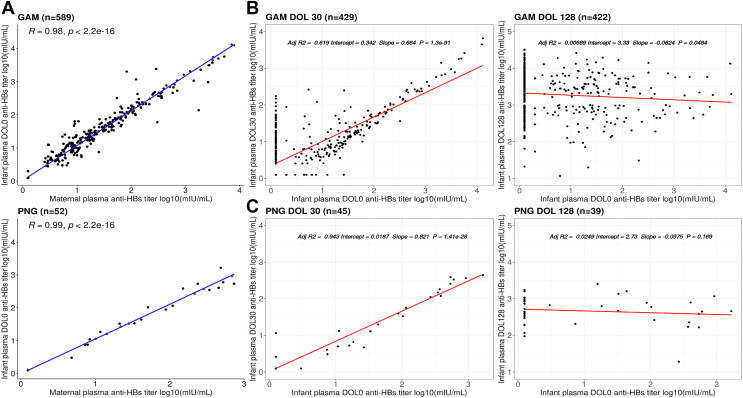
Relationship of maternal and infant plasma anti-HBs titers. **A,** Pearson correlation of anti-HBs titers (mIU/mL) assessed in maternal plasma following delivery and in neonatal plasma following birth at DOL0 among GAM (n = 589) and PNG (n = 52) maternal-infant paired samples. **B,** Univariate linear regression results predicting anti-HBs titers assessed in infants at DOL30 based on anti-HBs titers assessed in infants at DOL0. **C,** Univariate linear regression results predicting anti-HBs titers assessed in infants at DOL128 based on anti-HBs titers assessed in infants at DOL0. These analyses were performed on maternal-infant pairs with concordant detectable (≥2.5 mIU/mL) and nondetectable (<2.5 mIU/mL) anti-HBs titers at DOL0.

A significantly strong positive linear correlation was found between anti-HBs titers measured in mothers at time of delivery and anti-HBs titers measured in infant plasma at DOL0 in both cohorts (GAM *r* = 0.98, *P* < .001; PNG *r* = 0.99, *P* < .001) (Fig 4, *A*).

Univariate linear regression models predicting infant DOL30 and DOL128 anti-HBs titers from maternal-derived infant DOL0 anti-HBs titers revealed a significantly strong positive linear relationship between DOL0 and DOL30 anti-HBs titers in both cohorts (Fig 4, *B* and *C*). Specifically, in the GAM cohort, anti-HBs titers assessed in neonates at DOL0 account for 56% of the variance in DOL30 anti-HBs titers (GAM: adjusted *R*^2^ = 0.56, *P* < .001). In the PNG cohort, anti-HBs titers assessed in neonates at DOL0 explained 94.5% of the variability in DOL30 anti-HBs titers (PNG: adjusted *R*^2^ = 0.945, *P* < .001). Both cohorts showed no significance when predicting infant DOL128 anti-HBs titers based on infant plasma DOL0 anti-HBs titers (GAM: adjusted *R*^2^ = 0.006, *P* = .056; PNG: adjusted *R*^2^ = 0.024, *P* = .172) (Fig 4, *B* and *C*), by which time point all infants had received 3 doses of HBV. In addition, a weak correlation was found between infant anti-HBs titers at DOL30 and DOL128 (GAM *r* = 0.08, *P* = .073; PNG *r* = −0.26, *P* = .12) (see Fig E5 in this article’s Online Repository at www.jaci-global.org). From this analysis, we infer that maternally derived titers at DOL0 shape the infants’ humoral response at DOL30, but by DOL128 vaccine-induced titers are driving the infants’ response.

## Discussion

This report provides an overview of baseline clinical characteristics and exploratory analysis of HBV humoral immunogenicity in 2 randomized cohorts of infants in GAM and PNG, as part of a unique neonatal biology study that prospectively compared individual and combined immunization with BCG and HBV at birth. Enrollment captured representative samples of local ethnic groups in both cohorts, including the Mandinka in GAM and Eastern Highland groups in PNG. This design facilitated the study of neonatal immunogenicity across diverse geographic, genetic, and epigenetic backgrounds. Similar baseline characteristics and growth trajectories across vaccine groups indicate successful randomization and support robust between-group comparisons with minimal confounding.

Exploratory analyses show that Gambian infants who first received BCG vaccination at birth followed by HBV vaccination at DOL7 had higher DOL30 anti-HBs titers, which supports previous reports that early BCG vaccination can serve an adjuvant function to enhance immunogenicity of subsequent vaccines.^27^ However, larger studies should be considered to investigate this relationship further. This study also highlighted the influence of placental anti-HBs titer transfer on infant HBV immunogenicity in these distinct cohort populations. When we examined anti-HBs titers measured in infants at DOL30 compared with anti-HBs titers measured in the same infants at DOL0, we observed a strong positive linear relationship in both cohorts. However, when we consider infant DOL128 anti-HBs titers, a weak linear relationship with anti-HBs titers measured in infants at DOL0 (and DOL30) was observed in both cohorts, indicating that the effect of transplacentally acquired MatAbs measured in infant plasma at DOL0 is transient. Although MatAbs provide initial passive immunity to the newborns, they may suppress the neonate’s primary humoral response to immunization.^28^ As MatAbs levels decline, the infant’s immune system independently responds to HBV.^13^^,^^29^ Temporal dynamics of immune responses in neonates should be considered in the context of MatAbs when refining the timing and design of vaccination schedules to maximize efficacy. Irrespective of the influence of MatAbs, all Gambian infants (n = 459) and PNG infants (n = 96) with paired anti-HBs titers across all 3 time points (DOL0, DOL30, and DOL128) reached the hepatitis B CoP by DOL128 after completion of the primary series (Fig 3, *E*). This observation supports previous findings that the primary HBV series induces seroprotection in 98% of healthy term infants.^15^

The immunologic trends presented in this study also support previous publications from the same cohort populations, in which a cell-mediated immunity analysis demonstrated increased populations of antigen-specific polyfunctional CD154^+^IL-2^+^TNF-α^+^ CD4^+^ T cells following a single birth dose of HBV in a proportion of infants.^30^ In addition, polyfunctional T cells were positively correlated with serum antibody titers following the birth dose (DOL30) and completion of the 3-dose primary HBV series (DOL128).

A key strength of the EPIC-002 study is its prospective, randomized design across geographically distinct populations, with the ability to validate differences in infant immunogenicity during the first 128 days of life. Optimized blood collection procedures and rigorous data management ensured high-quality data while paired longitudinal samples from the same study participant enabled detailed insights into immune response dynamics. Despite logistical and technical challenges of studying newborns in resource-limited settings across multiple sites, the study implemented protocols designed to address the unique constraints of neonatal research^10^^,^^31^, ^32^, ^33^, ^34^, ^35^, ^36^, ^37^ including limited blood sample volumes available,^6^ sample transport, and cold-chain maintenance. These hurdles were addressed through community partnership and collaboration with local health authorities and standardized site procedures, highlighting a level of trust with the local community and the clinical study. Although our study focused on vaccine immunogenicity, we recognized that early-life environmental factors may influence immune priming during the first week of life. Future systems-level studies integrating these factors with molecular data will be important to provide biological insight into mechanisms by which vaccines induce protection in newborns and for refining early-life vaccination strategies. The high retention rate of participants through the critical early months of life reflected the high-quality efforts of the local recruiting staff and added to the reliability of the data and observed correlations. Although the study has several strengths, it is not without limitations. A key constraint is the lack of follow-up samples for the delayed vaccination group in the Gambian cohort, which precludes a complete longitudinal response assessment in this group. The absence of detailed maternal medical and vaccination records limited our ability to assess maternal health impacts on infant immunologic outcomes. However, this was partially accounted for by having only healthy, hepatitis B–, tuberculosis-, and HIV-negative mothers enrolled in the study as defined by the inclusion and exclusion criteria. Moreover, in some cases, discrepant values in the lower limit of quantification for anti-HBs titer measurements necessitated the removal of these data points for cleaner analysis though this reduced the overall sample size. Despite these limitations, successful randomization minimized confounding, ensuring that our findings regarding neonatal immunogenicity are robust and generalizable.

This study demonstrates the feasibility of recruiting prospective randomized cohorts in resource-limited settings to capture clinical data and samples amenable to downstream multiomic systems biology. It provides valuable insights into the determinants of neonatal vaccine immunogenicity, including the timing of vaccinations and impact of MatAbs, which should be considered as potential modifiers of vaccine immunogenicity and may account for observed variability in the magnitude and duration of vaccine-induced immune responses. Further research integrating clinical, immunologic, and systems biology data is warranted to characterize relationships between maternal factors, infant immune development, and vaccine immunogenicity to optimize early-life immunization strategies. The next steps of the National Institutes of Health / National Institutes of Allergy and Infectious Diseases Human Immunology Project Consortium–supported EPIC-002 study will implement a multiomic approach to define molecular biomarkers predictive of HBV immunogenicity in relation to co-administration of BCG with HBV, ontogeny, and the impact of MatAbs.

## Disclosure statement

This work is supported by grants from the National Institutes of Health / National Institute of Allergy and Infectious Diseases Human Immunology Project Consortium grant (grant no. U19 AI118608), Immune Development in Early Life grant (grant no. U19 AI168643), and Precision Vaccines Program, Department of Pediatrics, Boston Children’s Hospital. Core funding to the MRC Unit, The Gambia, facilitated recruitment (MC UP A900/1122). A.A. is supported in part by a National Institute of Allergy and Infectious Diseases Mentored Clinical Scientist Development Award (1K08AI168487). Funding for O.A.O.'s role in this project was provided by the Boston Children's Hospital T32 Neonatal Research Training Program from the National Institute of Health (T32-HD098061; PI: Kourembanas; from 2023-2024).

Disclosure of potential conflict of interest: O. Levy is a named inventor on patents held by Boston Children’s Hospital related to vaccine adjuvants and human *in vitro* systems that model immune responses, is a cofounder of *ARMR Sciences*, and has received sponsored research from and served as a consultant to GlaxoSmithKline. R. Ben-Othman is a current employee of RAN BioLinks. O. T. Idoko is a current employee of Sanofi, holds stock and stock options and is named on 2 patents. Her contributions were completed before this role. The sponsors had no role in the design, execution, interpretation, or writing of the study. The rest of the authors declare that they have no relevant conflicts of interest.

Data sharing: Clinical and experimental data sets have been made publicly available to the scientific community through ImmPort (accession nos. SDY1538 and SDY2584; www.immport.org).

## Supplementary data

Fig E1

Fig E2

Fig E3

Fig E4

Fig E5

Appendix

Table E1
