# Supplementary figures and images for: A randomized prospective study of neonatal hepatitis B vaccine immunogenicity in The Gambia and Papua New Guinea
Source: J Allergy Clin Immunol Glob. 2026 Feb 6;5(3):100653. doi: 10.1016/j.jacig.2026.100653 (PMC12968416; doi:10.1016/j.jacig.2026.100653)

# AFRICA

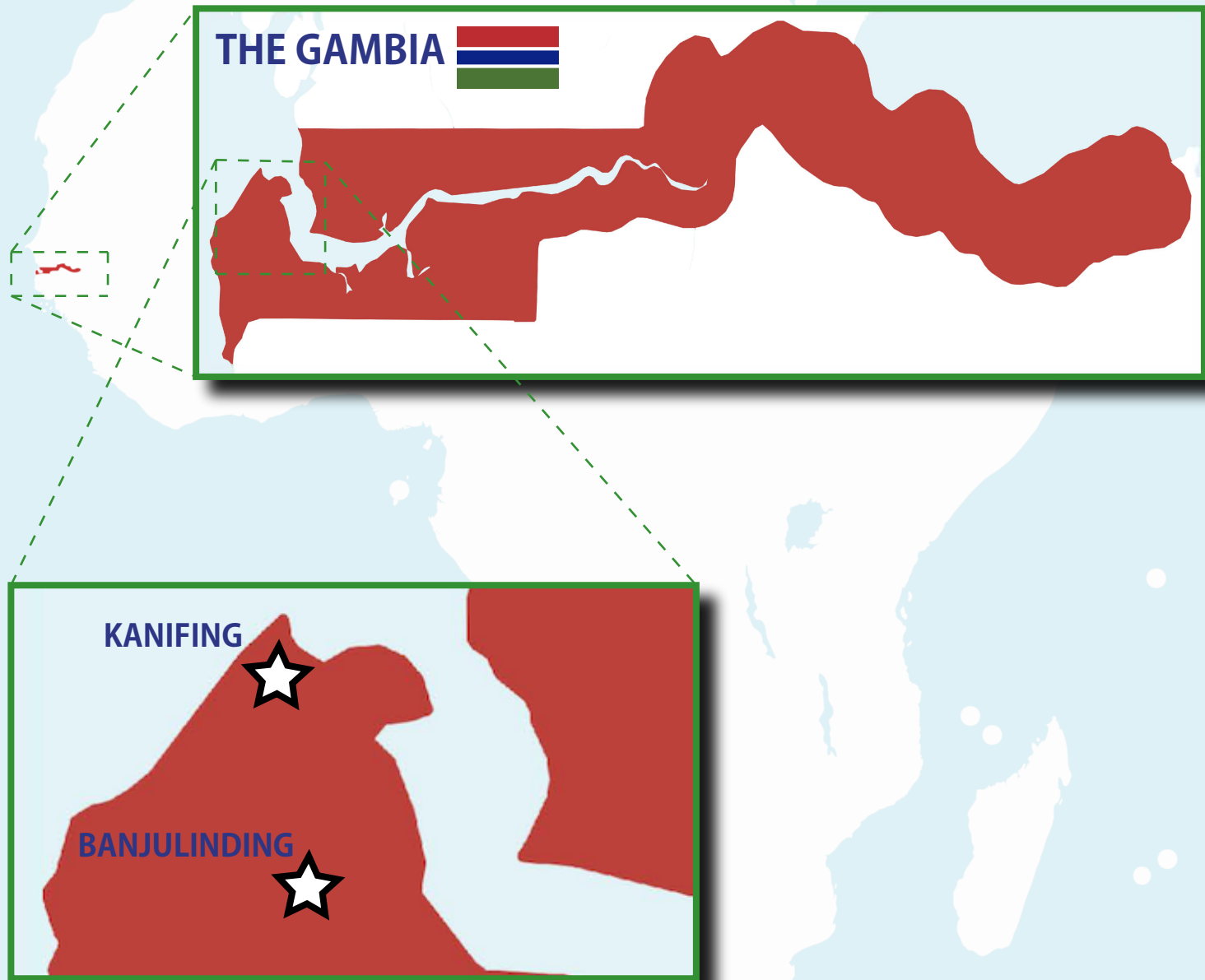

# PAPUA NEW GUINEA

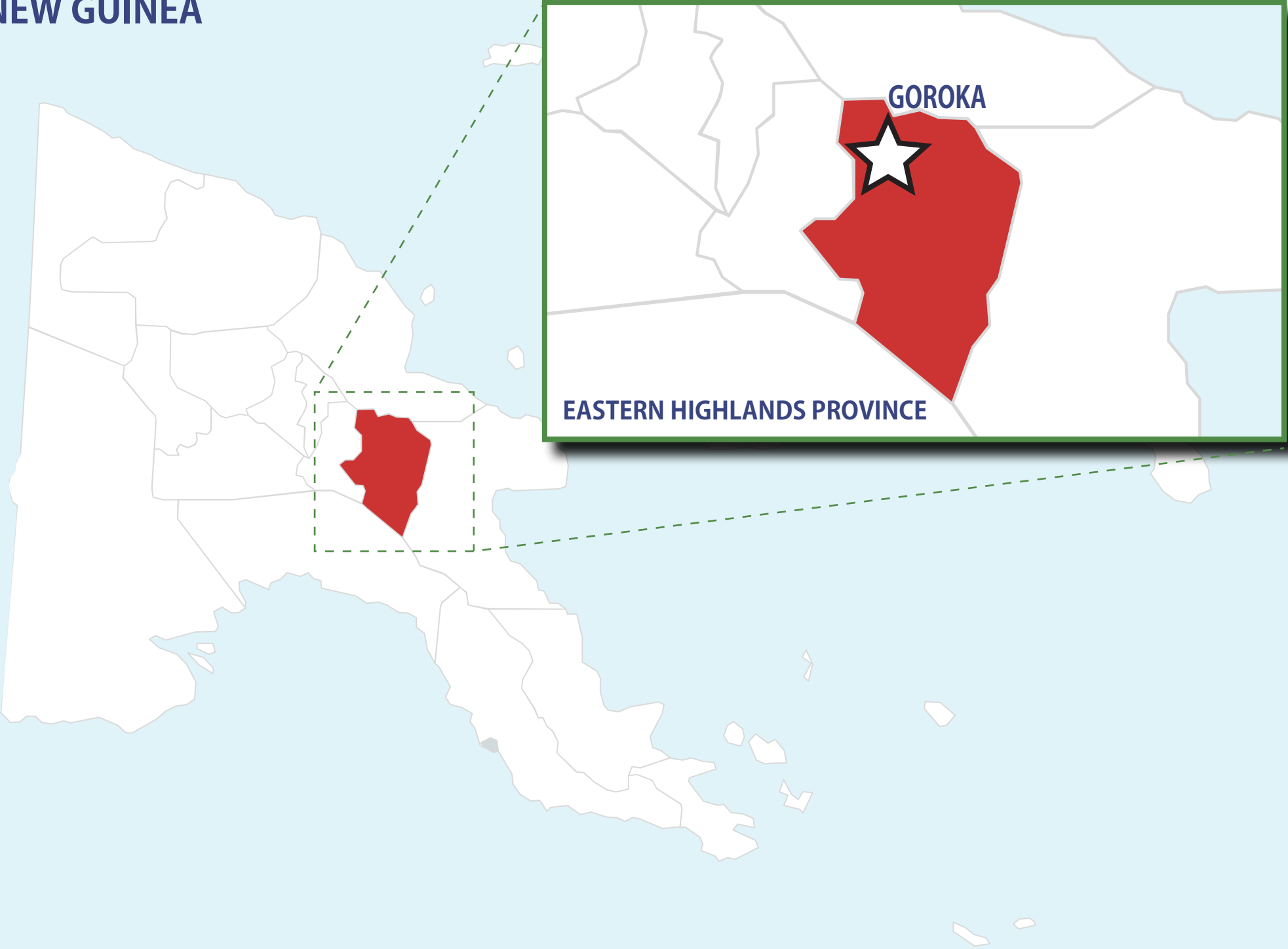

Supplement: Fig E1 [file mmc1.pdf]

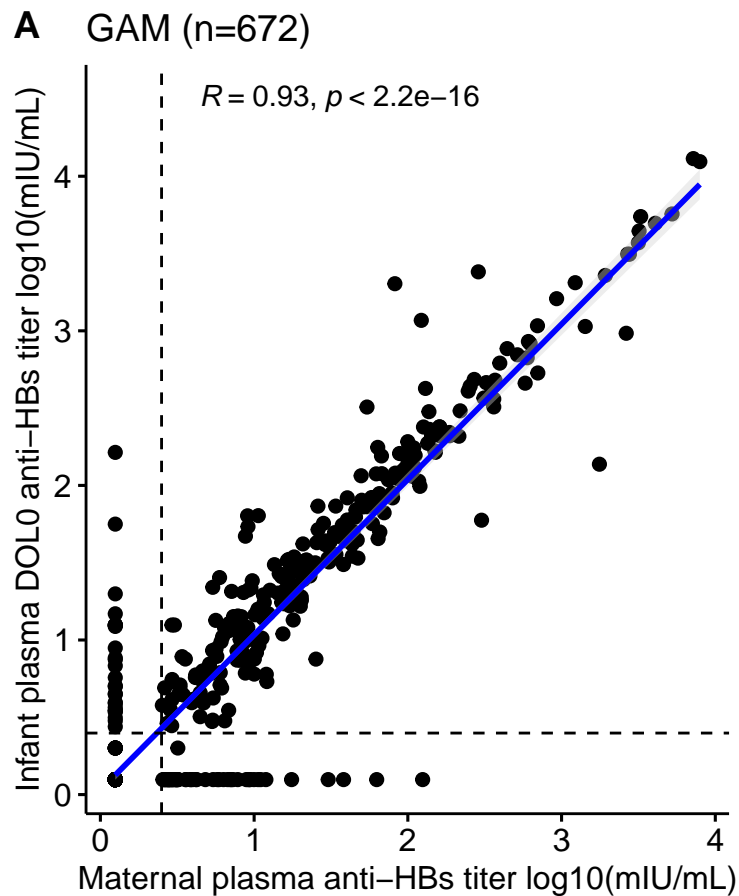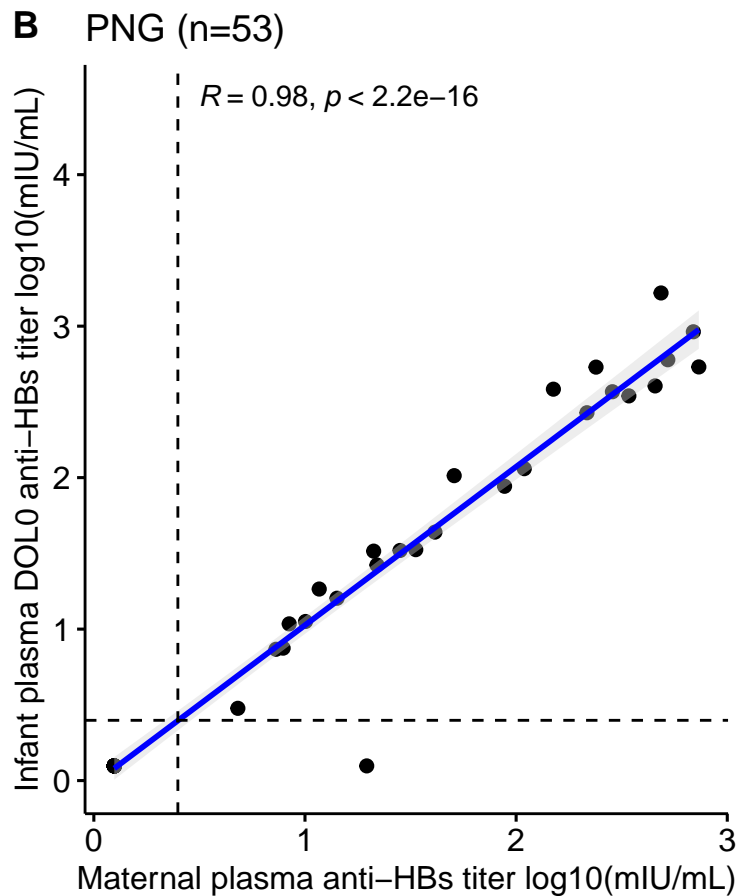

Supplement: Fig E2 [file mmc2.pdf]

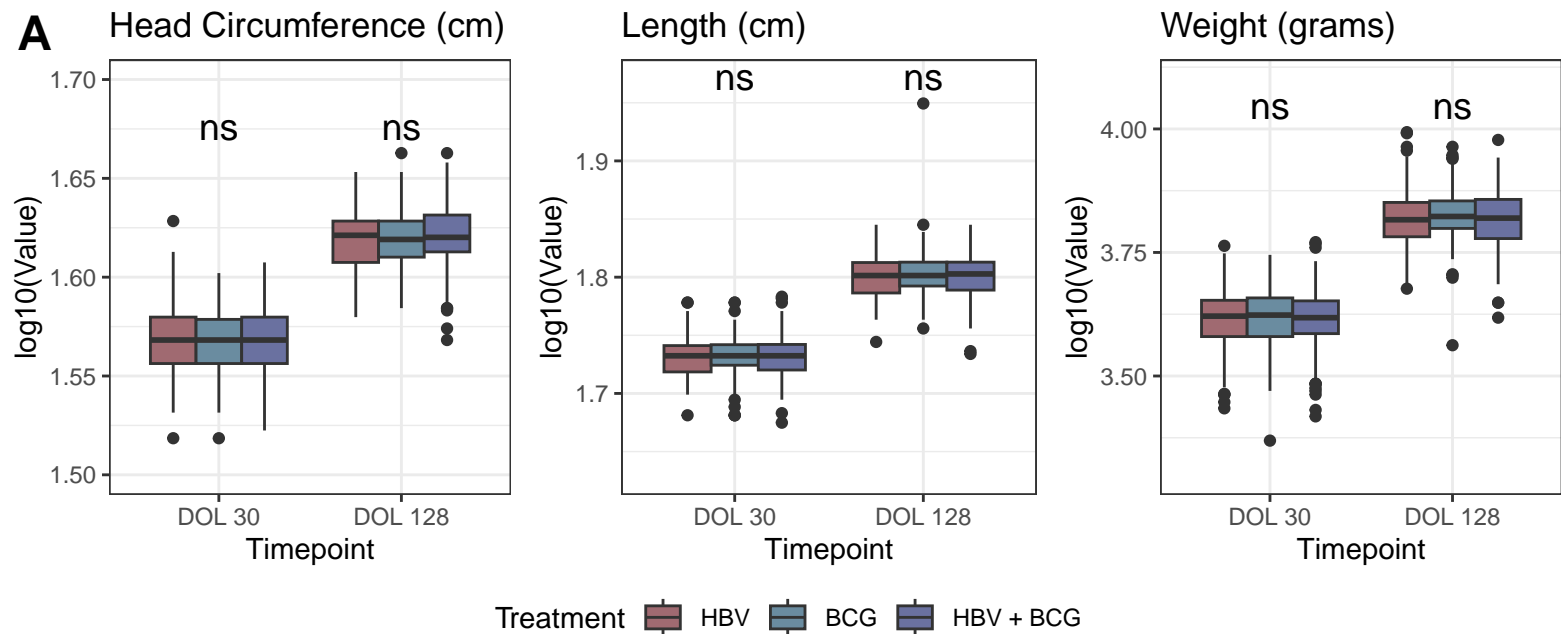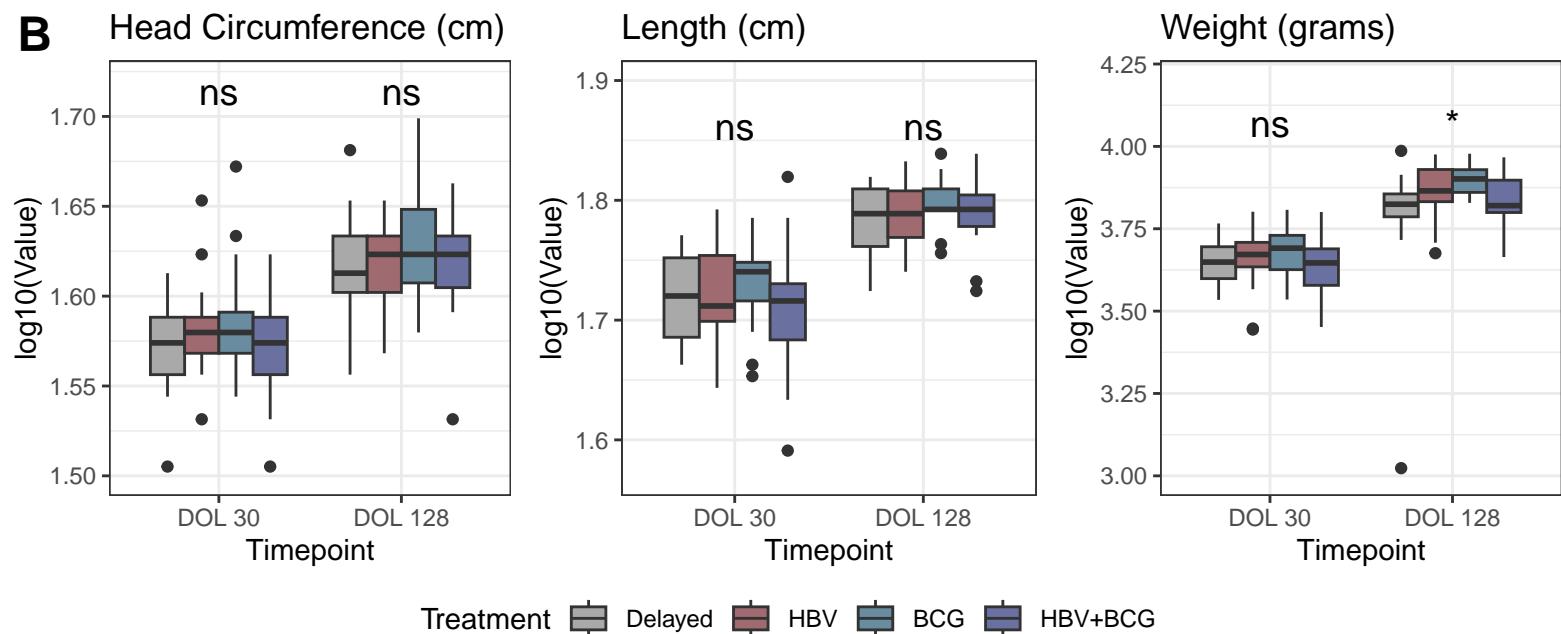

Supplement: Fig E3 [file mmc3.pdf]

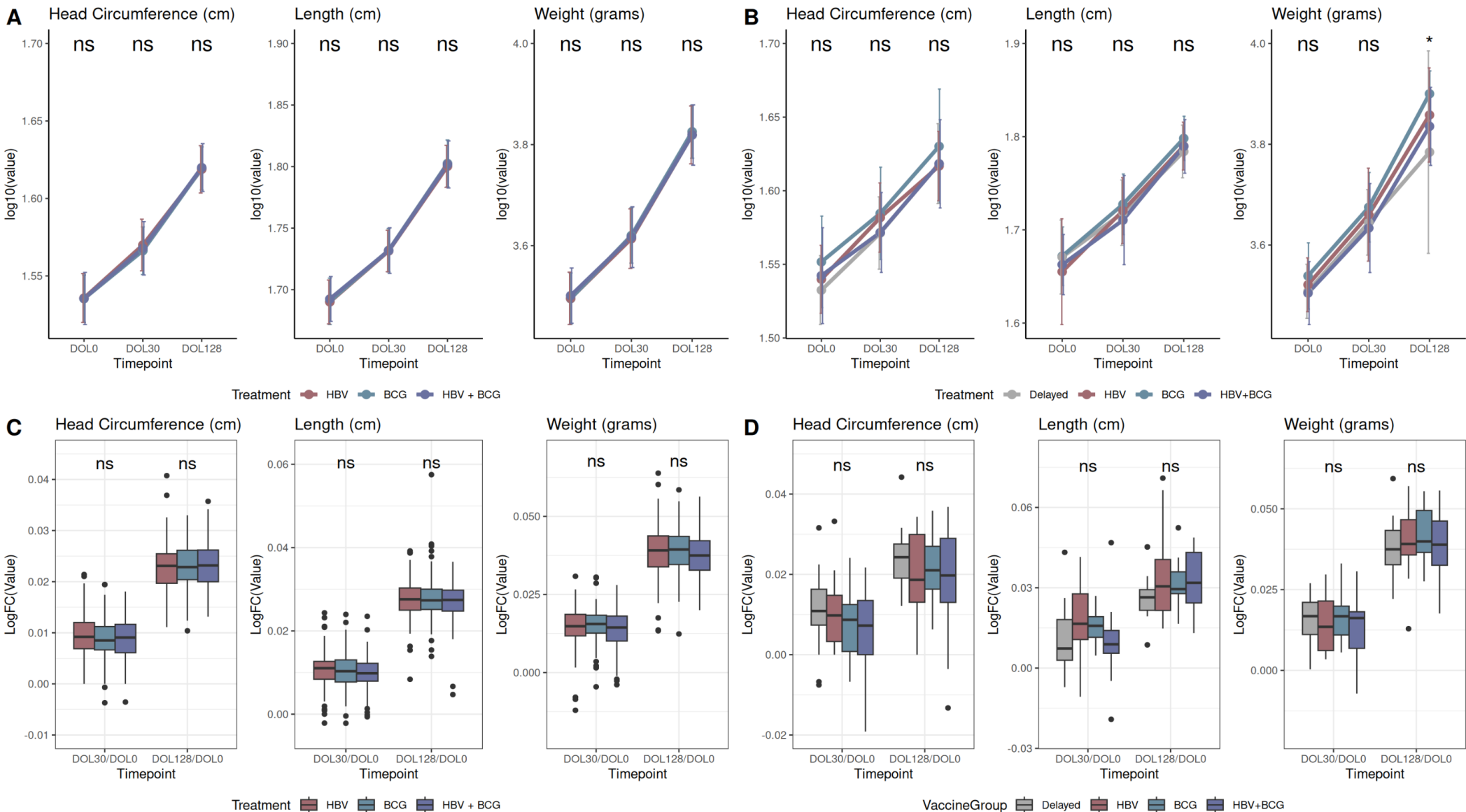

Supplement: Fig E4 [file mmc4.pdf]

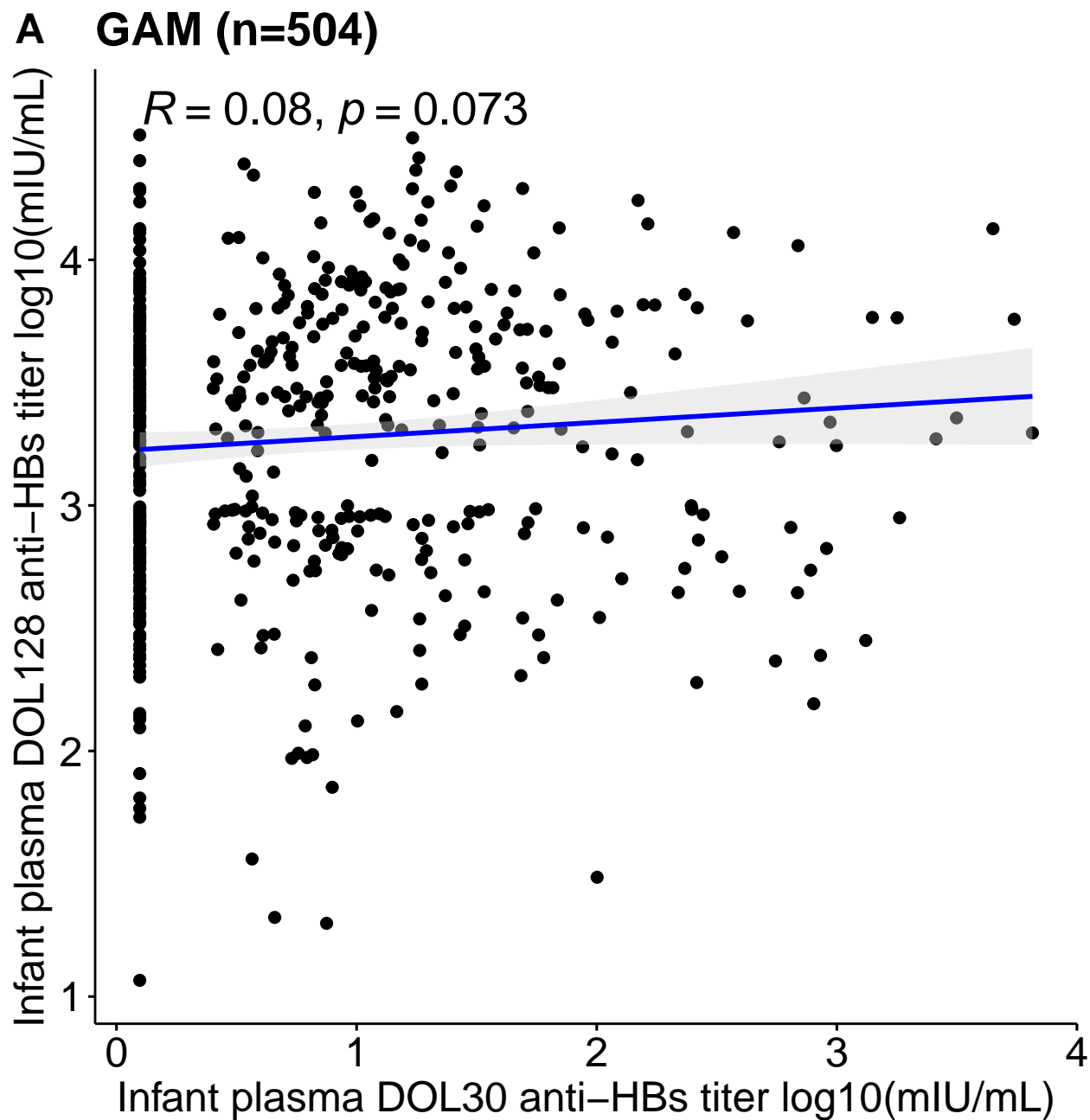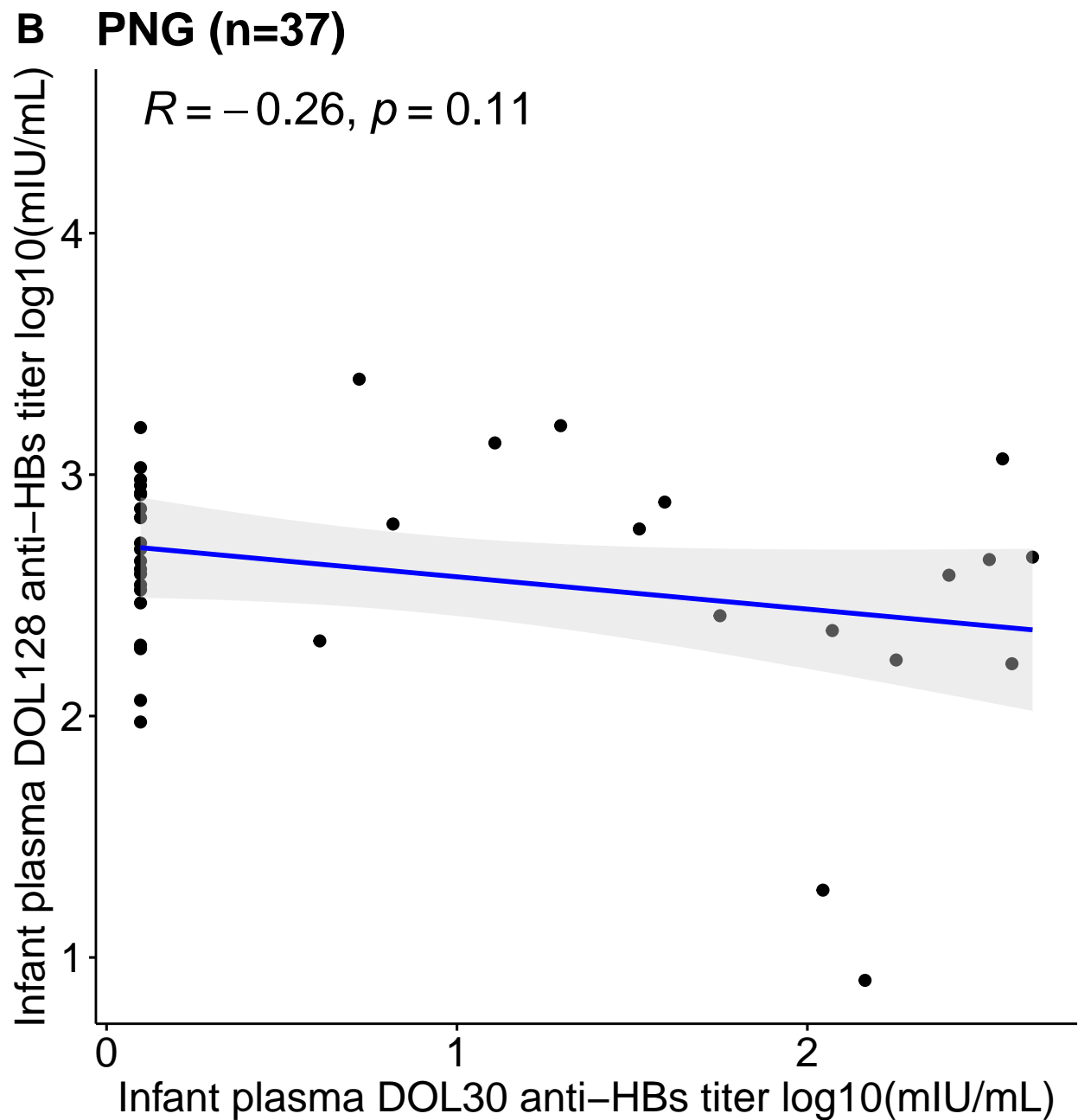

Supplement: Fig E5 [file mmc5.pdf]
