# Supplementary material for: A randomized prospective study of neonatal hepatitis B vaccine immunogenicity in The Gambia and Papua New Guinea
Source: J Allergy Clin Immunol Glob. 2026 Feb 6;5(3):100653. doi: 10.1016/j.jacig.2026.100653 (PMC12968416; doi:10.1016/j.jacig.2026.100653)
Supplement: Appendix [file mmc6.docx]

**Appendix 1**

**Supplementary Materials:**

**Figure E1.** **Maps of Expanded Program on Immunization Consortium (EPIC) study.** **(A)** MRC The Gambia (main cohort) and **(B)** IMR Papua New Guinea (validation cohort) study sites.

**Figure E2. Relationship of all paired maternal-infant anti-HBs titers.** Pearson correlation of all anti-HBs titers collected at DOL0 among **(A)** Gambian maternal-infant pairs (n=672) and **(B)** PNG maternal-infant pairs (n=53). The dotted horizontal and vertical lines represent the lower limit of detection (2.5 mIU/mL) for the anti-HBs titer assay.

**Figure E3.** **Comparison of mean growth measures across vaccine groups indicates successful randomization in GAM and PNG.** Kruskal-Wallis rank sum global significance markers are shown for differences in mean growth measures for matched DOL30 (V3) and DOL128 (V4) samples in **(A)** Gambian infants (n=519) and **(B)** PNG infants (n=97) across vaccine groups (HBV, BCG, HBV+BCG, Delayed) (ns=not significant, *p<0.05). The Delayed vaccine group in the Gambian infant cohort was not followed to DOL30 or DOL128.

**Figure E4. Longitudinal changes in infant growth measures across vaccine randomization groups.** Kruskal-Wallis rank sum significance markers are shown for differences in logfc(DOL30/DOL0) and logfc(DOL128/DOL0) infant growth measures for matched DOL0, DOL30, and DOL128 samples in **(A)** Gambian infants (n=531) and **(B)** PNG infants (n=97) across vaccine groups (HBV, BCG, HBV+BCG, Delayed). Kruskal-Wallis rank sum significance markers are shown for differences in mean repeated infant growth measures for matched DOL0 (V1), DOL30 (V3) and DOL128 (V4) samples in **(C)** Gambian infants (n=531) and **(D)** PNG infants (n=97) across vaccine groups. Mean log10 concentrations and standard error bars are shown for each group at each timepoint. The Delayed vaccine group in the Gambian infant cohort was not followed to DOL30 or DOL128. (ns=not significant, *p<0.05)

**Figure E5. Lack of correlation between infant anti-HBs titers at DOL30 and DOL128.** Pearson correlation of anti-HBs titers (mIU/mL) assessed in infant plasma at DOL30 and DOL128 among **(A)** Gambian (n=504) and **(B)** PNG (n=37) paired infant samples.
