# Supplementary material for: A randomized prospective study of neonatal hepatitis B vaccine immunogenicity in The Gambia and Papua New Guinea
Source: J Allergy Clin Immunol Glob. 2026 Feb 6;5(3):100653. doi: 10.1016/j.jacig.2026.100653 (PMC12968416; doi:10.1016/j.jacig.2026.100653)
Supplement: Table E1 [file mmc7.docx]

**Table E1.** Final diagnoses captured for hospitalized Gambian infants (n=59) stratified by vaccine groups.

|  | **HBV**  **(n=20)** | **BCG (n=12)** | **HBV+BCG (n=16)** | **Delayed (n=11)** |
| --- | --- | --- | --- | --- |
| **Final hospital diagnosis, No. (%)** |  |  |  |  |
| **Acute gastroenteritis** | 0 (0) | 3 (25) | 1 (6) | 0 (0) |
| **Amniotic fluid aspiration** | 1 (5) | 0 (0) | 0 (0) | 0 (0) |
| **Apparent life-threatening event** | 1 (5) | 0 (0) | 0 (0) | 0 (0) |
| **Bacterial lower respiratory tract infection** | 2 (10) | 0 (0) | 0 (0) | 0 (0) |
| **Bronchiolitis** | 3 (15) | 2 (17) | 1 (6) | 1 (9) |
| **Bullous impetigo** | 1 (5) | 0 (0) | 0 (0) | 0 (0) |
| **Dehydration fever** | 0 (0) | 0 (0) | 1 (6) | 0 (0) |
| **Early onset neonatal sepsis** | 5 (25) | 1 (8) | 7 (44) | 1 (9) |
| **Gastric outlet obstruction** | 0 (0) | 0 (0) | 0 (0.0) | 1 (9) |
| **Generalized impetigo and pneumonia** | 0 (0) | 1 (8) | 0 (0.0) | 0 (0) |
| **Late onset neonatal sepsis** | 2 (10) | 0 (0) | 1 (6) | 1 (9) |
| **Neonatal jaundice** | 4 (20) | 3 (25) | 1 (6) | 2 (18) |
| **Omphalitis** | 0 (0) | 0 (0) | 0 (0) | 1 (9) |
| **Overwhelming sepsis** | 0 (0) | 0 (0) | 0 (0) | 1 (9) |
| **Pneumonia** | 1 (5) | 0 (0) | 1 (6) | 3 (27) |
| **Primary failure to thrive** | 0 (0) | 0 (0) | 1 (6) | 0 (0) |
| **Severe bronchopneumonia** | 0 (0) | 1 (8) | 0 (0) | 0 (0) |
| **Streptococcus pneumonia meningitis** | 0 (0) | 1 (8) | 0 (0) | 0 (0) |
| **Sudden infant death syndrome (SIDS)** | 0 (0) | 0 (0) | 1 (6) | 0 (0) |
| **Viral infection** | 0 (0) | 0 (0) | 1 (6) | 0 (0) |

**Note:** Column percentages may not total to 100 due to rounding to the nearest whole number.

**Table E2A.** Wilcoxon pairwise comparison results for Figure 3A.

| **Cohort** | **DOL** | **Outcome** | **group1** | **group2** | **n1** | **n2** | **Wilcoxon Test Statistic (W)** | **P-value** | **Wilcoxon Effect Size (r)** |
| --- | --- | --- | --- | --- | --- | --- | --- | --- | --- |
| GAM | DOL0 | log10(infant anti-HBs titer (mIU/mL)) | HBV | BCG | 98 | 95 | 5114 | 0.237 | 0.095 |
| GAM | DOL0 | log10(infant anti-HBs titer (mIU/mL)) | HBV | HBV + BCG | 98 | 110 | 5834.5 | 0.306 | 0.075 |
| GAM | DOL0 | log10(infant anti-HBs titer (mIU/mL)) | BCG | HBV + BCG | 95 | 110 | 5126.5 | 0.817 | 0.021 |
| GAM | DOL30 | log10(infant anti-HBs titer (mIU/mL)) | HBV | BCG | 142 | 134 | 9864 | 0.598 | 0.057 |
| GAM | DOL30 | log10(infant anti-HBs titer (mIU/mL)) | HBV | HBV + BCG | 142 | 142 | 10304 | 0.749 | 0.050 |
| GAM | DOL30 | log10(infant anti-HBs titer (mIU/mL)) | BCG | HBV + BCG | 134 | 142 | 9300.5 | 0.748 | 0.016 |
| GAM | DOL128 | log10(infant anti-HBs titer (mIU/mL)) | HBV | BCG | 152 | 140 | 10636.5 | 0.997 | 0.017 |
| GAM | DOL128 | log10(infant anti-HBs titer (mIU/mL)) | HBV | HBV + BCG | 152 | 158 | 12442 | 0.583 | 0.009 |
| GAM | DOL128 | log10(infant anti-HBs titer (mIU/mL)) | BCG | HBV + BCG | 140 | 158 | 11565 | 0.497 | 0.032 |

**Table E2B.** Wilcoxon pairwise comparison results for Figure 3B.

| **Cohort** | **DOL** | **Outcome** | **group1** | **group2** | **n1** | **n2** | **Wilcoxon Test Statistic (W)** | **P-value** | **Wilcoxon Effect Size (r)** |
| --- | --- | --- | --- | --- | --- | --- | --- | --- | --- |
| PNG | DOL0 | log10(infant anti-HBs titer (mIU/mL)) | Delayed | HBV | 14 | 14 | 0.874 | 0.874 | 0.035 |
| **PNG** | **DOL0** | **log10(infant anti-HBs titer (mIU/mL))** | **Delayed** | **BCG** | **14** | **7** | **44** | **0.037** | **0.081** |
| PNG | DOL0 | log10(infant anti-HBs titer (mIU/mL)) | Delayed | HBV + BCG | 14 | 12 | 103 | 0.347 | 0.192 |
| PNG | DOL0 | log10(infant anti-HBs titer (mIU/mL)) | HBV | BCG | 14 | 7 | 47 | 0.913 | 0.033 |
| PNG | DOL 0 | log10(infant anti-HBs titer (mIU/mL)) | BCG | HBV + BCG | 7 | 12 | 50 | 0.536 | 0.155 |
| PNG | DOL30 | log10(infant anti-HBs titer (mIU/mL)) | Delayed | HBV | 15 | 17 | 115.5 | 0.664 | 0.080 |
| PNG | DOL30 | log10(infant anti-HBs titer (mIU/mL)) | Delayed | BCG | 15 | 13 | 108 | 0.65 | 0.091 |
| PNG | DOL30 | log10(infant anti-HBs titer (mIU/mL)) | Delayed | HBV + BCG | 15 | 13 | 101 | 0.892 | 0.030 |
| PNG | DOL30 | log10(infant anti-HBs titer (mIU/mL)) | HBV | BCG | 17 | 13 | 140 | 0.229 | 0.225 |
| PNG | DOL30 | log10(infant anti-HBs titer (mIU/mL)) | HBV | HBV + BCG | 17 | 13 | 126 | 0.536 | 0.118 |
| PNG | DOL30 | log10(infant anti-HBs titer (mIU/mL)) | BCG | HBV + BCG | 13 | 13 | 77 | 0.724 | 0.075 |
| **PNG** | **DOL128** | **log10(infant anti-HBs titer (mIU/mL))** | **Delayed** | **HBV** | **17** | **16** | **201** | **0.019** | **0.408** |
| PNG | DOL128 | log10(infant anti-HBs titer (mIU/mL)) | Delayed | BCG | 17 | 12 | 142 | 0.08 | 0.329 |
| PNG | DOL128 | log10(infant anti-HBs titer (mIU/mL)) | Delayed | HBV + BCG | 17 | 18 | 164 | 0.732 | 0.061 |
| PNG | DOL128 | log10(infant anti-HBs titer (mIU/mL)) | HBV | BCG | 16 | 12 | 84 | 0.599 | 0.105 |
| **PNG** | **DOL128** | **log10(infant anti-HBs titer (mIU/mL))** | **HBV** | **HBV + BCG** | **16** | **18** | **86** | **0.046** | **0.343** |
| PNG | DOL128 | log10(infant anti-HBs titer (mIU/mL)) | BCG | HBV + BCG | 12 | 18 | 75 | 0.172 | 0.255 |

**Table E2C.** Wilcoxon pairwise comparison results for Figure 3C.

| **Cohort** | **Day** | **Outcome** | **group1** | **group2** | **n1** | **n2** | **Wilcoxon Test Statistic (W)** | **P-value** | **Wilcoxon Effect Size (r)** |
| --- | --- | --- | --- | --- | --- | --- | --- | --- | --- |
| GAM | D1 | log10(infant DOL30 anti-HBs titer (mIU/mL)) | HBV | BCG | 48 | 40 | 1107.5 | 0.218 | 0.183 |
| GAM | D1 | log10(infant DOL30 anti-HBs titer (mIU/mL)) | HBV | HBV + BCG | 48 | 47 | 1260 | 0.329 | 0.119 |
| GAM | D1 | log10(infant DOL30 anti-HBs titer (mIU/mL)) | BCG | HBV + BCG | 40 | 47 | 920 | 0.868 | 0.049 |
| GAM | D3 | log10(infant DOL30 anti-HBs titer (mIU/mL)) | HBV | BCG | 47 | 45 | 1042.5 | 0.91 | 0.007 |
| GAM | D3 | log10(infant DOL30 anti-HBs titer (mIU/mL)) | HBV | HBV + BCG | 47 | 45 | 904.5 | 0.234 | 0.099 |
| GAM | D3 | log10(infant DOL30 anti-HBs titer (mIU/mL)) | BCG | HBV + BCG | 45 | 45 | 872.5 | 0.26 | 0.102 |
| GAM | D7 | log10(infant DOL30 anti-HBs titer (mIU/mL)) | HBV | BCG | 47 | 49 | 1150 | 0.994 | 0.022 |
| GAM | D7 | log10(infant DOL30 anti-HBs titer (mIU/mL)) | HBV | HBV + BCG | 47 | 50 | 1263.5 | 0.525 | 0.058 |
| GAM | D7 | log10(infant DOL30 anti-HBs titer (mIU/mL)) | BCG | HBV + BCG | 49 | 50 | 1292.5 | 0.639 | 0.008 |

**Table E2D.** Wilcoxon pairwise comparison results for Figure 3D.

| **Cohort** | **Treatment** | **Outcome** | **group1** | **group2** | **n1** | **n2** | **Wilcoxon Test Statistic (W)** | **P-value** | **Wilcoxon Effect Size (r)** |
| --- | --- | --- | --- | --- | --- | --- | --- | --- | --- |
| GAM | HBV | log10_ConcentrationActual | DOL1 | DOL3 | 52 | 54 | 1519.5 | 0.467 | 0.071 |
| GAM | HBV | log10_ConcentrationActual | DOL1 | DOL7 | 52 | 50 | 1269 | 0.838 | 0.021 |
| GAM | HBV | log10_ConcentrationActual | DOL3 | DOL7 | 54 | 50 | 1181.5 | 0.274 | 0.107 |
| GAM | BCG | log10_ConcentrationActual | DOL1 | DOL3 | 53 | 50 | 1138.5 | 0.22 | 0.121 |
| **GAM** | **BCG** | **log10_ConcentrationActual** | **DOL1** | **DOL7** | **53** | **59** | **1220** | **0.046** | **0.189** |
| GAM | BCG | log10_ConcentrationActual | DOL3 | DOL7 | 50 | 59 | 1333 | 0.39 | 0.083 |
| GAM | HBV + BCG | log10_ConcentrationActual | DOL1 | DOL3 | 53 | 50 | 1049.5 | 0.07 | 0.179 |
| GAM | HBV + BCG | log10_ConcentrationActual | DOL1 | DOL7 | 53 | 55 | 1204 | 0.12 | 0.150 |
| GAM | HBV + BCG | log10_ConcentrationActual | DOL3 | DOL7 | 50 | 55 | 1429.5 | 0.729 | 0.034 |

**Table E3.** Kruskal-Wallis rank sum test results for Figure E3.

| **Cohort** | **Anthropometric measure (y)** | **Timepoint** | **DF** | **Kruskal-Wallis chi-square statistic** | **P-value** |
| --- | --- | --- | --- | --- | --- |
| GAM | Head Circumference (cm) | DOL30 | 2 | 2.475 | 0.290 |
| GAM | Head Circumference (cm) | DOL128 | 2 | 0.528 | 0.768 |
| GAM | Length (cm) | DOL30 | 2 | 0.290 | 0.865 |
| GAM | Length (cm) | DOL128 | 2 | 1.254 | 0.534 |
| GAM | Weight (g) | DOL30 | 2 | 0.436 | 0.804 |
| GAM | Weight (g) | DOL128 | 2 | 2.032 | 0.362 |
| PNG | Head Circumference (cm) | DOL30 | 3 | 2.643 | 0.450 |
| PNG | Head Circumference (cm) | DOL128 | 3 | 0.822 | 0.844 |
| PNG | Length (cm) | DOL30 | 3 | 2.401 | 0.493 |
| PNG | Length (cm) | DOL128 | 3 | 1.713 | 0.634 |
| PNG | Weight (g) | DOL30 | 3 | 3.508 | 0.320 |
| **PNG** | **Weight (g)** | **DOL128** | **3** | **9.915** | **0.019** |

**Table E4.** Kruskal-Wallis rank sum test results for Figure E4 comparing infant anthropometric measurements across vaccine groups at each timepoint.

| **Cohort** | **Anthropometric measure (y)** | **Timepoint** | **DF** | **Kruskal-Wallis chi-square statistic** | **P-value** |
| --- | --- | --- | --- | --- | --- |
| GAM | Head Circumference (cm) | DOL0 | 3 | 0.062 | 0.970 |
| GAM | Head Circumference (cm) | DOL30 | 3 | 2.470 | 0.290 |
| GAM | Head Circumference (cm) | DOL128 | 3 | 0.528 | 0.768 |
| GAM | Weight (g) | DOL0 | 3 | 1.430 | 0.489 |
| GAM | Weight (g) | DOL30 | 3 | 0.436 | 0.804 |
| GAM | Weight (g) | DOL128 | 3 | 2.032 | 0.362 |
| GAM | Length (cm) | DOL0 | 3 | 2.022 | 0.364 |
| GAM | Length (cm) | DOL30 | 3 | 0.290 | 0.865 |
| GAM | Length (cm) | DOL128 | 3 | 1.254 | 0.534 |
| PNG | Head Circumference (cm) | DOL0 | 3 | 5.400 | 0.145 |
| PNG | Head Circumference (cm) | DOL30 | 3 | 2.643 | 0.450 |
| PNG | Head Circumference (cm) | DOL128 | 3 | 0.822 | 0.844 |
| PNG | Weight (g) | DOL0 | 3 | 5.490 | 0.1392 |
| PNG | Weight (g) | DOL30 | 3 | 3.508 | 0.320 |
| **PNG** | **Weight (g)** | **DOL128** | **3** | **9.915** | **0.019** |
| PNG | Length (cm) | DOL0 | 3 | 2.024 | 0.567 |
| PNG | Length (cm) | DOL30 | 3 | 2.401 | 0.493 |
| PNG | Length (cm) | DOL128 | 3 | 1.713 | 0.634 |
| GAM | Head Circumference (cm) | DOL30/DOL0 | 2 | 3.132 | 0.209 |
| GAM | Head Circumference (cm) | DOL128/DOL0 | 2 | 0.507 | 0.776 |
| GAM | Length (cm) | DOL30/DOL0 | 2 | 3.441 | 0.179 |
| GAM | Length (cm) | DOL128/DOL0 | 2 | 0.493 | 0.781 |
| GAM | Weight (g) | DOL30/DOL0 | 2 | 2.953 | 0.228 |
| GAM | Weight (g) | DOL128/DOL0 | 2 | 4.865 | 0.088 |
| PNG | Head Circumference (cm) | DOL30/DOL0 | 3 | 2.020 | 0.568 |
| PNG | Head Circumference (cm) | DOL128/DOL0 | 3 | 1.395 | 0.707 |
| PNG | Length (cm) | DOL30/DOL0 | 3 | 5.265 | 0.153 |
| PNG | Length (cm) | DOL128/DOL0 | 3 | 3.231 | 0.357 |
| PNG | Weight (g) | DOL30/DOL0 | 3 | 1.038 | 0.792 |
| PNG | Weight (g) | DOL128/DOL0 | 3 | 1.965 | 0.578 |

**Table E5.** Kruskal-Wallis rank sum test results for Figure 3E.

| **Cohort** | **Timepoint** | **DF** | **Kruskal-Wallis chi-square statistic** | **P-value** |
| --- | --- | --- | --- | --- |
| GAM | DOL0 | 2 | 0.222 | 0.895 |
| GAM | DOL30 | 2 | 0.208 | 0.901 |
| GAM | DOL128 | 2 | 0.511 | 0.774 |
| PNG | DOL0 | 3 | 6.305 | 0.098 |
| PNG | DOL30 | 3 | 3.736 | 0.291 |
| PNG | DOL128 | 3 | 7.727 | 0.052 |

**Table E6.** Wilcoxon rank-sum test statistics and effect size for anthropometric measures shown in Figure E3.

| **DOL** | **Anthropometric Measure (y)** | **group1** | **group2** | **n1** | **n2** | **Wilcoxon Test Statistic (W)** | **P-value** | **Wilcoxon Effect Size (r)** |
| --- | --- | --- | --- | --- | --- | --- | --- | --- |
| DOL 30 | Weight (grams) | Delayed | HBV | 22 | 22 | 197.5 | 0.30 | 0.157 |
| DOL 30 | Weight (grams) | Delayed | BCG | 22 | 21 | 172 | 0.16 | 0.219 |
| DOL 30 | Weight (grams) | Delayed | HBV+BCG | 22 | 22 | 265 | 0.60 | 0.081 |
| DOL 30 | Weight (grams) | HBV | BCG | 22 | 21 | 221.5 | 0.83 | 0.035 |
| DOL 30 | Weight (grams) | HBV | HBV+BCG | 22 | 22 | 287 | 0.30 | 0.159 |
| DOL 30 | Weight (grams) | BCG | HBV+BCG | 21 | 22 | 296 | 0.12 | 0.241 |
| DOL 128 | Weight (grams) | Delayed | HBV | 18 | 16 | 101 | 0.144 | 0.254 |
| **DOL 128** | **Weight (grams)** | **Delayed** | **BCG** | **18** | **12** | **35** | **0.001** | **0.564** |
| DOL 128 | Weight (grams) | Delayed | HBV+BCG | 18 | 18 | 137.5 | 0.448 | 0.129 |
| DOL 128 | Weight (grams) | HBV | BCG | 16 | 12 | 70.5 | 0.246 | 0.224 |
| DOL 128 | Weight (grams) | HBV | HBV+BCG | 16 | 18 | 174 | 0.309 | 0.178 |
| **DOL 128** | **Weight (grams)** | **BCG** | **HBV+BCG** | **12** | **18** | **164** | **0.019** | **0.432** |
| DOL 30 | Length (cm) | Delayed | HBV | 22 | 22 | 235.0 | 0.878 | 0.025 |
| DOL 30 | Length (cm) | Delayed | BCG | 22 | 21 | 199.5 | 0.449 | 0.117 |
| DOL 30 | Length (cm) | Delayed | HBV+BCG | 22 | 22 | 268.0 | 0.548 | 0.092 |
| DOL 30 | Length (cm) | HBV | BCG | 22 | 21 | 197.0 | 0.414 | 0.127 |
| DOL 30 | Length (cm) | HBV | HBV+BCG | 22 | 22 | 263.5 | 0.620 | 0.076 |
| DOL 30 | Length (cm) | BCG | HBV+BCG | 21 | 22 | 301.5 | 0.088 | 0.262 |
| DOL 128 | Length (cm) | Delayed | HBV | 18 | 16 | 130.5 | 0.652 | 0.080 |
| DOL 128 | Length (cm) | Delayed | BCG | 18 | 12 | 77.5 | 0.200 | 0.238 |
| DOL 128 | Length (cm) | Delayed | HBV+BCG | 18 | 18 | 150.5 | 0.726 | 0.061 |
| DOL 128 | Length (cm) | HBV | BCG | 16 | 12 | 76.5 | 0.373 | 0.173 |
| DOL 128 | Length (cm) | HBV | HBV+BCG | 16 | 18 | 139.5 | 0.890 | 0.027 |
| DOL 128 | Length (cm) | BCG | HBV+BCG | 12 | 18 | 128.0 | 0.404 | 0.156 |
| **Supplementary Table 6 (continued)** | | | | | | | | |
| **DOL** | **Anthropometric Measure (y)** | **group1** | **group2** | **n1** | **n2** | **Wilcoxon Test Statistic (W)** | **P-value** | **Wilcoxon Effect Size (r)** |
| DOL 30 | Head circumference (cm) | Delayed | HBV | 22 | 22 | 193.0 | 0.244 | 0.177 |
| DOL 30 | Head circumference (cm) | Delayed | BCG | 22 | 21 | 185.0 | 0.263 | 0.172 |
| DOL 30 | Head circumference (cm) | Delayed | HBV+BCG | 22 | 22 | 241.5 | 1.000 | 0.002 |
| DOL 30 | Head circumference (cm) | HBV | BCG | 22 | 21 | 230.0 | 0.990 | 0.004 |
| DOL 30 | Head circumference (cm) | HBV | HBV+BCG | 22 | 22 | 290.5 | 0.249 | 0.176 |
| DOL 30 | Head circumference (cm) | BCG | HBV+BCG | 21 | 22 | 276.0 | 0.274 | 0.169 |
| DOL 128 | Head circumference (cm) | Delayed | HBV | 18 | 16 | 142.0 | 0.958 | 0.012 |
| DOL 128 | Head circumference (cm) | Delayed | BCG | 18 | 12 | 89.5 | 0.442 | 0.144 |
| DOL 128 | Head circumference (cm) | Delayed | HBV+BCG | 18 | 18 | 152.0 | 0.761 | 0.053 |
| DOL 128 | Head circumference (cm) | HBV | BCG | 16 | 12 | 79.5 | 0.453 | 0.146 |
| DOL 128 | Head circumference (cm) | HBV | HBV+BCG | 16 | 18 | 136.0 | 0.794 | 0.048 |
| DOL 128 | Head circumference (cm) | BCG | HBV+BCG | 12 | 18 | 121.5 | 0.579 | 0.105 |
